# LY6D is crucial for lipid accumulation and inflammation in nonalcoholic fatty liver disease

**DOI:** 10.1038/s12276-023-01033-w

**Published:** 2023-07-03

**Authors:** Jibeom Lee, Hyeonhui Kim, Yun-Won Kang, Yumin Kim, Moon-young Park, Ji-Hong Song, Yunju Jo, Tam Dao, Dongryeol Ryu, Junguee Lee, Chang-Myung Oh, Sangkyu Park

**Affiliations:** 1 Department of Biomedical Science and Engineering, Gwangju Institute of Science and Technology, Gwangju, Korea; 2 Graduate School of Medical Science, Brain Korea 21 Project, Yonsei University College of Medicine, Seoul, Korea; 3 Department of Molecular Cell Biology, Sungkyunkwan University (SKKU) School of Medicine, Suwon, Korea; 4 Department of Pathology, St Mary’s Hospital, the Catholic University of Korea, Daejeon, Korea; 5 Department of Precision Medicine, Yonsei University Wonju College of Medicine, Wonju, Korea; 6 Mitohormesis Research Center, Yonsei University Wonju College of Medicine, Wonju, Gangwon-do Korea

**Keywords:** Non-alcoholic fatty liver disease, Transcriptomics

## Abstract

Nonalcoholic fatty liver disease (NAFLD) is a serious metabolic disorder characterized by excess fat accumulation in the liver. Over the past decade, NAFLD prevalence and incidence have risen globally. There are currently no effective licensed drugs for its treatment. Thus, further study is required to identify new targets for NAFLD prevention and treatment. In this study, we fed C57BL6/J mice one of three diets, a standard chow diet, high-sucrose diet, or high-fat diet, and then characterized them. The mice fed a high-sucrose diet had more severely compacted macrovesicular and microvesicular lipid droplets than those in the other groups. Mouse liver transcriptome analysis identified lymphocyte antigen 6 family member D (*Ly6d*) as a key regulator of hepatic steatosis and the inflammatory response. Data from the Genotype-Tissue Expression project database showed that individuals with high liver Ly6d expression had more severe NAFLD histology than those with low liver Ly6d expression. In AML12 mouse hepatocytes, *Ly6d* overexpression increased lipid accumulation, while *Ly6d* knockdown decreased lipid accumulation. Inhibition of *Ly6d* ameliorated hepatic steatosis in a diet-induced NAFLD mouse model. Western blot analysis showed that Ly6d phosphorylated and activated ATP citrate lyase, which is a key enzyme in de novo lipogenesis. In addition, RNA- and ATAC-sequencing analyses revealed that *Ly6d* drives NAFLD progression by causing genetic and epigenetic changes. In conclusion, *Ly6d* is responsible for the regulation of lipid metabolism, and inhibiting *Ly6d* can prevent diet-induced steatosis in the liver. These findings highlight *Ly6d* as a novel therapeutic target for NAFLD.

## Liver disease: Regulatory gene tied to fat accumulation

A gene implicated in regulating fat accumulation in the liver offers a potential new target for the treatment and prevention of non-alcoholic fatty liver disease (NAFLD). A research team from South Korea led by Chang-Myung Oh (Gwangju Institute of Science and Technology) and Sangkyu Park (Yonsei University Wonju College of Medicine) showed that liver tissue taken from mice with diet-induced obesity had elevated expression of *Ly6d*, a gene also found to be more active in the livers of people with NAFLD. By manipulating levels of *Ly6d* in mouse liver cells and in mouse models of NAFLD, the researchers showed that, among other regulatory functions, the protein encoded by *Ly6d* activates a key enzyme involved in fat synthesis. Drugs targeting this gene or its protein product could help combat excess fat build-up in the liver.

## Introduction

Nonalcoholic fatty liver disease (NAFLD) encompasses a broad range of liver diseases, from benign fatty liver to nonalcoholic steatohepatitis (NASH). NASH is characterized by steatosis with hepatocyte ballooning and is associated with progression to liver fibrosis and HCC^1,2^. It is also significantly associated with nonhepatic metabolic diseases, such as type-2 diabetes, chronic kidney disease, cardiovascular vascular disease, and gastrointestinal cancer^3,4^. Over the past decade, the worldwide prevalence and incidence of NAFLD have increased significantly^5^. During this period, there has been significant progress in understanding the pathophysiology of NAFLD, and many risk factors have been identified for this condition^6,7^. However, there are currently no effective licensed drugs for its treatment^7^. Thus, there is an urgent need for further studies to identify novel targets for NAFLD prevention and treatment.

Obesity is a major risk factor for NAFLD and has shown significant associations with other NAFLD risk factors, such as metabolic syndrome, type-2 diabetes, and dyslipidemia^6^. Thus, researchers have developed a diet-induced animal model and used it to elucidate NAFLD pathophysiology^8^. A high-fat diet (HFD), with fat content ranging from 32–60%, has been commonly used to induce obesity and metabolic diseases in mice^9,10^. Western diets enriched in both fat and carbohydrates (HSD), especially fructose and sucrose^11^, are also used for generating models with diet-induced obesity.

Lymphocyte antigen 6 family member D (LY6D) is a membrane-bound protein^12^. In humans, *LY6D* is expressed in esophageal and skin tissues and associated with cancers^13^. Mouse *Ly6d* mRNA is mainly found in immune cells, such as B cells, T cells, and dendritic cells^13^, and is used as a cell surface marker for immune cell subsets^14^. However, whether and how Ly6D contributes to NAFLD development remains unclear.

In the present study, we found that Ly6d expression was significantly higher in the livers of HFD- and HSD-fed mice than in mice fed a standard chow diet (SCD). We aimed to investigate the functional role of Ly6d in hepatic steatosis. To address this, we used hepatocyte cell lines and a mouse model of diet-induced NAFLD. Based on a multidisciplinary approach consisting of bulk RNA sequencing (RNA-seq), ATAC sequencing (ATAC-seq), and single-cell RNA sequencing (scRNA-seq), we found that Ly6d was significantly associated with NAFLD progression in both humans and mice. In vitro and in vivo studies have revealed that Ly6d regulates hepatic steatosis by means of phosphorylation of ATP citrate lyase (Acly). These findings suggest that Ly6d could serve as a potential therapeutic target for NAFLD.

## Materials and methods

### Animal models

Seven-week-old C57BL/6J male mice were fed a SCD for 1 week for adaptation. For diet-induced disease studies, 8-week-old mice were randomly divided into three groups and separated into independent cages. The three mouse groups were then placed on either a SCD, HSD (D12079B, Research Diets, New Brunswick, NJ 08901 USA), or HFD (D12492, Research Diets) for 19 weeks until they were 27 weeks old. The mice were fed experimental diets and water ad libitum. All mice were on a 12 h/12 h day/night cycle in a specific pathogen-free mouse facility.

### Glucose and insulin tolerance tests

At 24 weeks of age, after 17 weeks of diet intervention, all mice fasted for at least 16 h. Then, they were weighed, and the glucose concentration in their blood was measured through the mouse tail vein. This value was considered the glucose concentration at 0 min. Then, two grams of glucose per kilogram of each mouse was injected into the abdominal cavity of the mice, and the blood glucose concentration was measured at 15, 30, 60, 90, and 120 min thereafter. For the insulin tolerance tests, the mice were intraperitoneally injected with insulin (0.75 U/kg) after 4 h of fasting.

### Histological analysis

Mouse liver and epididymal white adipose tissues were fixed in 10% neutral buffered formalin solution (Sigma‒Aldrich, Gyeonggi-do, South Korea) before being embedded in paraffin. Deparaffinized 5-mm-thick tissue sections were rehydrated and stained with hematoxylin and eosin. Picrosirius red was also used to stain the liver tissue sections.

### Cell culture and maintenance

Mouse hepatocyte AML12 cells were maintained in DMEM/F12 (Welgene, Gyeongsangbuk-do, South Korea) with fetal bovine serum, dexamethasone, and insulin transferrin solution (I3146, Sigma) at 37 °C in a 5% CO_2_-containing incubator. The concentration of fructose was chosen to be four times higher (69.96 mM) than the concentration of glucose precontained in the complete medium (17.49 mM) to create a condition similar to that in the in vivo mouse model.

### Quantitative real-time PCR

RNA was extracted from the cells using TRIzol™ reagent (15596026, Invitrogen) and resuspended. The mRNA was then used to synthesize cDNA using a cDNA Synthesis Kit (4368814, Applied Biosystems, Walthan, MA, USA). The synthesized cDNA was diluted at a ratio of 1:10 and used to analyze mRNA expression by means of quantitative real-time PCR using SYBR^®^ Green Master Mix (Q5600, GenDEPOT, Katy, TX, USA).

### Western blot analysis

AML12 cells were lysed in radioimmunoprecipitation lysis buffer containing 1% protease and phosphatase inhibitor cocktail. Protein concentrations were determined using a Qubit-iT™ Protein Assay Kit (Invitrogen). Equal amounts of protein were separated using 8–12% SDS‒PAGE and then transferred to polyvinylidene fluoride membranes. After blocking with 5% skim milk for 1 h at room temperature, the membranes were probed with specific primary antibodies, including rabbit anti-P-ACLY/Ser455 (1:1000, 4331, Cell Signaling Technology, Danvers, Massachusetts, USA), anti-ACLY (1:1000, 4332, Cell Signaling Technology), anti-P-acetyl-CoA carboxylase (ACC)/Ser79 (1:2000, 3661, Cell Signaling Technology), anti-ACC (1:2000, 3676, Cell Signaling Technology), anti-FAS (1:2000, 3180, Cell Signaling Technology), anti-P-AMPKα/Thr172 (1:1000, 2531, Cell Signaling Technology), anti-AMPKα (1:1000, 5831, Cell Signaling Technology), anti-α-tubulin (1:2500, 2144, Cell Signaling Technology), anti-β-actin (1:2500, 12262, Cell Signaling Technology), and mouse anti-SREBP-1 (1:2000, 365513, Santa Cruz Biotechnology, Heidelberg. Germany) overnight at 4 °C and then probed with the corresponding secondary antibodies for 1 h at room temperature. Signals were developed using a Clarity™ Western ECL Substrate Kit (Bio-Rad, Hercules, CA, USA). The specific protein bands were visualized using the LuminoGraph II System (ATTO, Tokyo, Japan).

### RNA-seq and data analysis

The R program (version 4.1.0) was used to analyze and visualize the RNA-seq data, except for part of the preprocessing pipeline at trimming and alignment, where STAR (version 2.7.9) was used. We utilized the DESeq2 package (version 1.32.0) as a step for differentially expressed gene (DEG) analysis. To visualize the analyzed data, we used pheatmap, EnhancedVolcano, and the ggplot2 package in R. The DEG results of each group comparison were analyzed in terms of adjusted *p* values obtained using the Benjamini–Hochberg tool. All data are expressed as the mean ± SEM.

The gene list for heatmap expression was processed using a gene set enrichment analysis (GSEA) database search. The EnrichR web-based server was used to analyze the upregulated DEGs. To identify the upstream regulator, Ingenuity^®^ Pathway Analysis software (IPA^®^, QIAGEN, Redwood City, CA, USA; www.qiagen.com/ingenuity) was used to identify the DEGs in the *Ly6d* overexpression dataset. *P* values were calculated using Fisher’s exact test, with a significance cutoff of 0.05 and an activation *Z* score cutoff of ±2^15^. The data on the gene list of each biological pathway were accessed using the Molecular Signature Database (https://www.gsea-msigdb.org/gsea/index.jsp).

### ScRNA-seq

The count matrix was generated from the ‘Bioproduct_Stats.csv’ and ‘Sample_Tag_Calls.csv’ files from each group, following the instructions given in the BD Single-Cell Genomics Bioinformatics Handbook. For cell filtering, we excluded the cell index with the ‘Sample_Tag’ field value as ‘Undetermined’ and ‘Multiplet’. For gene filtering, only genes with the ‘Depth_Status’ field value as ‘pass’ in the experimental groups were used, except for the gene ‘*Ly6d*’. Finally, values in the ‘RSEC adjusted molecules’ field were used to create the count matrix. For the following processes, the ‘Seurat’ R package (version 4.2.0) was used.

Please see the Supplementary information for detailed information on the materials and processes used in this study.

## Results

### Diet-induced obesity leads to liver steatosis in mice

**Fig. 1:**
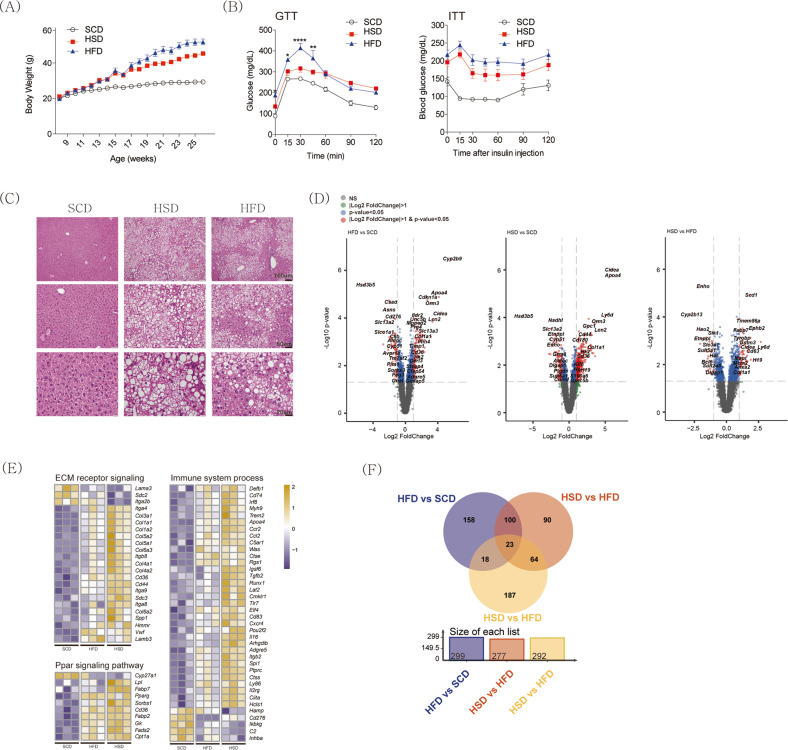
Diet-induced obesity and hepatic steatosis. C57BL6/J mice (*n* = 5) were fed one of three types of diets: SCD, HSD, or HFD. **A** Body weight changes during diet feeding. **B** Glucose and insulin tolerance tests. **C** Representative images of hematoxylin & eosin staining of liver tissues from each group. **D** Volcano plots showing the DEGs between each pair of groups: HFD-fed *vs*. SCD-fed; HSD-fed *vs*. SCD-fed; and HSD-fed *vs*. HFD-fed. **E** A heatmap of a set of genes associated with each GO biological process. **F** DEGs for the three comparisons are represented in the form of a Venn diagram. SCD, standard chow diet; HSD, high-sucrose high-fat diet; HFD, high-fat diet; DEGs, differentially expressed genes; GO, Gene Ontology. The average value displayed in **A**, **B**, and **C** is presented as the mean ± SED. The significance levels for the GTT and ITT indicated in **B** and **C** are *p < 0.05, **p < 0.01, ***p < 0.001, and ****p < 0.0001.

Measurement of body weights in the three groups of mice revealed that both the HSD- and HFD-fed groups had higher body weights than the SCD-fed group, although the HFD-fed mice gained weight faster than the HSD-fed group (Fig. 1A and Supplementary Fig. 1a). In the glucose tolerance test, the HFD-fed group displayed higher glucose concentrations in the blood than the HSD-fed group (Fig. 1B). In the insulin tolerance test, the glucose levels of the HSD-fed mice were altered in a manner similar to those of the HFD-fed mice (Fig. 1B). Compared to the SCD-fed group, both the HFD- and HSD-fed groups displayed condensed lipid droplet formation (Fig. 1C). The HSD-fed group had more severely compacted macro- and microvesicular lipid droplets, with the hepatic central vein and portal triad losing their casual development, as seen in histological images^16^. The number of inflammatory cells and crown-like structures in white adipose tissue was significantly higher in the HFD-fed group than in the SCD- and HSD-fed groups (Supplementary Fig. 1B).

To obtain a comprehensive explanation for diet-induced NAFLD progression, we performed RNA-seq analysis of samples from the livers of the SCD-, HSD-, and HFD-fed groups. To identify the key genes that alter the liver phenotype, we constructed three comparison groups: HFD-fed *vs*. SCD-fed, HSD-fed *vs*. SCD-fed, and HSD-fed *vs*. HFD-fed. Then, we carried out DEG analysis. Figure 1D shows the volcano plot representing the statistically significant DEGs in each comparison group, and the heatmaps of the identified DEGs between groups are shown in Supplementary Fig. 1c, d.

Compared to the SCD-fed group, the HFD- and HSD-fed groups displayed upregulated expression of cell death-inducing DFFA-like effector A (*Cidea*), stearoyl-CoA desaturase 1 (*Scd1*), collagen type I alpha 1 chain (*Col1a1*), and fatty acid-binding protein 7 (*Fabp7*), all of which have been linked to lipid metabolism, liver injury^17–20^, and HCC^17,21^. The HSD- and HFD-fed groups displayed higher expression levels of extracellular matrix receptor signaling, peroxisome proliferator-activated receptor (PPAR) signaling pathway, and immune system processes than the SCD-fed group (Fig. 1E and Supplementary Fig. 1e).

Then, we explored the genes that showed markedly different expression patterns between the HFD- and HSD-fed groups to identify a key driver that can elucidate the specific mechanisms of NAFLD development. Figure 1F shows a Venn diagram depicting the number of DEGs between the groups; a total of twenty-three DEGs were commonly expressed across the three groups (Supplementary Table 1).

### Upregulation of *LY6D* mRNA expression in the livers of humans with NAFLD

**Fig. 2:**
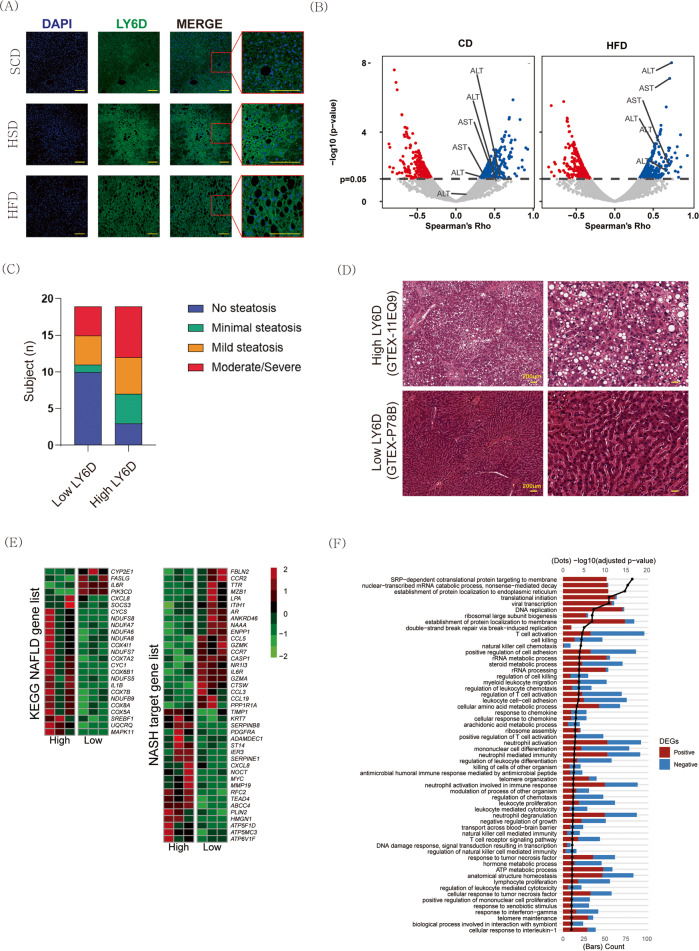
Positive associations between hepatic Ly6d expression and steatosis in both mice and humans. **A** Immunofluorescence images showing increased Ly6d expression in the livers of both HSD- and HFD-fed mice. Scale bar: 50 μm. **B** Volcano plots depicting the correlation (Spearman’s rho, x-axis) between hepatic *Ly6d* expression and all available phenotypes in the BXD mouse genetic reference population, as well as statistical significance levels (–log10[p value], y-axis). **C** Pathological findings in human liver tissues from the GTEx liver data, ranging from no steatosis to severe steatosis, with disease severity weighted in terms of *LY6d* expression levels. **D** Representative images of H&E staining of human liver tissues from the GTEx liver data. **E** Heatmaps of gene expression levels of liver DEGs related to NAFLD and NASH between subjects with high Ly6d expression and those with low Ly6d expression. The false discovery rate (FDR) was determined by the Benjamini‒Hochberg (BH) procedure. **F** Bar chart summarizing the pathway enrichment analysis of DEGs. HSD high-sucrose high-fat diet, HFD high-fat diet, H&E hematoxylin and eosin, NAFLD nonalcoholic fatty liver disease, NASH nonalcoholic steatohepatitis, DEGs differentially expressed genes.

Among the 23 DEGs, we focused on *Ly6D*, which was one of the most significantly upregulated DEGs (Fig. 1D). Ly6D expression was higher in the liver parenchyma of both the HSD- and HFD-fed groups than in the liver parenchyma of the SCD-fed group (Fig. 2A). To investigate the potential associations between *Ly6d* and liver disease, we analyzed the associations between *Ly6d* expression and mouse liver phenotypes of the BXD family using GeneNetwork. In the BXD murine reference panel^22^, *Ly6d* showed significant positive associations with aspartate transaminase and alanine aminotransferase, which are known serum biomarkers of hepatocellular injury (Fig. 2B)^22^.

Next, to determine the clinical relevance of Ly6d in the pathogenesis of NAFLD, we investigated whether there were histological and transcriptomic differences between the 20 subjects with the highest (high-LY6D group) and lowest (low-LY6D group) LY6D mRNA expression levels in the liver in the data from the GTEx project^23^. In line with the results in mice, severe hepatic steatosis was observed in the high-LY6D group (Fig. 2C, D). Transcriptomic analysis revealed that compared to the low-LY6D group, the high-LY6D group had different gene expression profiles for genes related to NAFLD and NASH. The risk genes driving the progression of NAFLD and profibrotic genes were upregulated in the high-LY6D group (Fig. 2E).

Figure 2F shows the enriched GO terms based on the DEGs between the two groups. The most significant GO terms in the high-LY6D group included “SRP-dependent cotranslational protein targeting the membrane”, “nuclear-transcriptional mRNA catabolic process, nonsense-mediated decay”, and “translational initiation”. GSEA revealed that LY6D-related genes were mainly enriched in adipocytokine signaling and carbohydrate metabolism (Supplementary Fig. 2a).

### Overexpression of *Ly6d* induces lipid accumulation in mouse hepatocytes

**Fig. 3:**
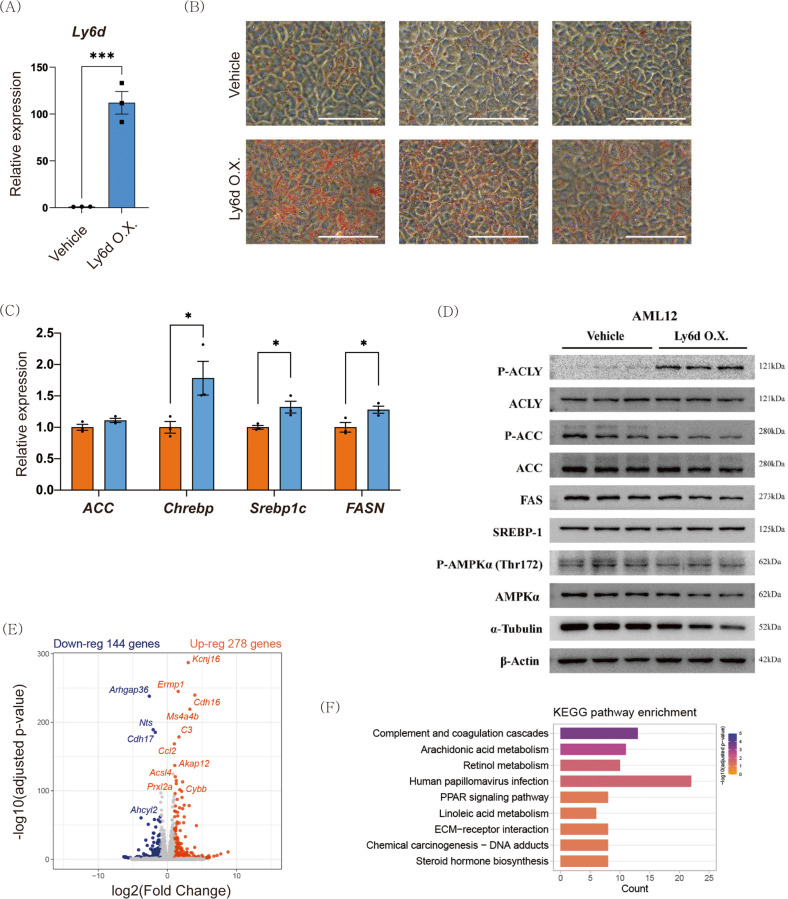
*Ly6d* overexpression induces lipid accumulation in hepatocytes. AML12 cells were transfected with either vehicle (Vehicle) or *Ly6d*-overexpressing plasmid (Ly6d O.X.). **A** *Ly6d* mRNA expression in the vehicle and Ly6d O.X. cell line. **B** Representative images of Oil Red O-stained AML12 cells. Scale bar: 50 μm. **C** Relative mRNA expression of essential genes involved in de novo lipogenesis. **D** Images of Western blots for phospho-ACLY, ACLY, phospho-ACC, ACC, FAS, SREBP-1, phospho-AMPKα, AMPKα, α-tubulin, and β-actin. **E** Volcano plot indicating DEGs between the Ly6d O.X. and vehicle cells. **F** Bar chart summarizing the enriched KEGG pathways (adjusted *p* < 0.05 and |log2-FC | > 1) in Ly6d O.X. cells *versus* vehicle cells. DEGs, differentially expressed genes. The false discovery rate (FDR) was determined by the Benjamini‒Hochberg (BH) procedure. The relative expression shown in **A** and **C** is presented as the mean ± SED, and the significance levels indicated in **B** and **C** are *p < 0.05, **p < 0.01, and ***p < 0.001.

To determine the role of *Ly6d* in hepatic steatosis and inflammation, we generated a *Ly6d*-overexpressing vector and transfected AML12 cells with it (Fig. 3A). Oil Red O staining revealed that there was more lipid deposition in *Ly6d*-overexpressing cells than in control cells (Fig. 3B). *Ly6d* overexpression also increased the mRNA expression of lipogenic genes (Fig. 3C). Western blot analysis revealed a significant increase in the phosphorylation of ACLY (Fig. 3D). ACLY is a critical enzyme involved in the synthesis of cytosolic acetyl-CoA, which is a necessary building block for de novo fatty acid synthesis^24^.

Next, we performed RNA-seq analysis to investigate the metabolic effects of *Ly6d* on mouse hepatocytes. This analysis identified 422 DEGs, as shown in the volcano plot (Fig. 3E). The most significantly upregulated DEGs were known candidates for NAFLD progression, such as potassium inwardly rectifying channel subfamily J member 16 (*Kcnj16*)^25^ and endoplasmic reticulum metallopeptidase 1 (*Ermp1*)^26^. Kyoto Encyclopedia of Genes and Genomes (KEGG) pathway analysis revealed that the top 3 significant pathways were “complement and coagulation cascades”, “arachidonic acid metabolism”, and “retinol metabolism”, all of which are associated with NAFLD development^27–29^. The DEGs between *Ly6d*-overexpressing cells and control cells were also enriched in pathways related to the “PPAR signaling pathway”, “ECM-receptor interaction”, and “steroid hormone biosynthesis” (Fig. 3F). GO analysis showed that the DEGs were involved in energy metabolism, inflammation, and fibrosis (Supplementary Fig. 2b).

### Chromatic accessibility is altered by *Ly6d* overexpression in mouse hepatocytes

**Fig. 4:**
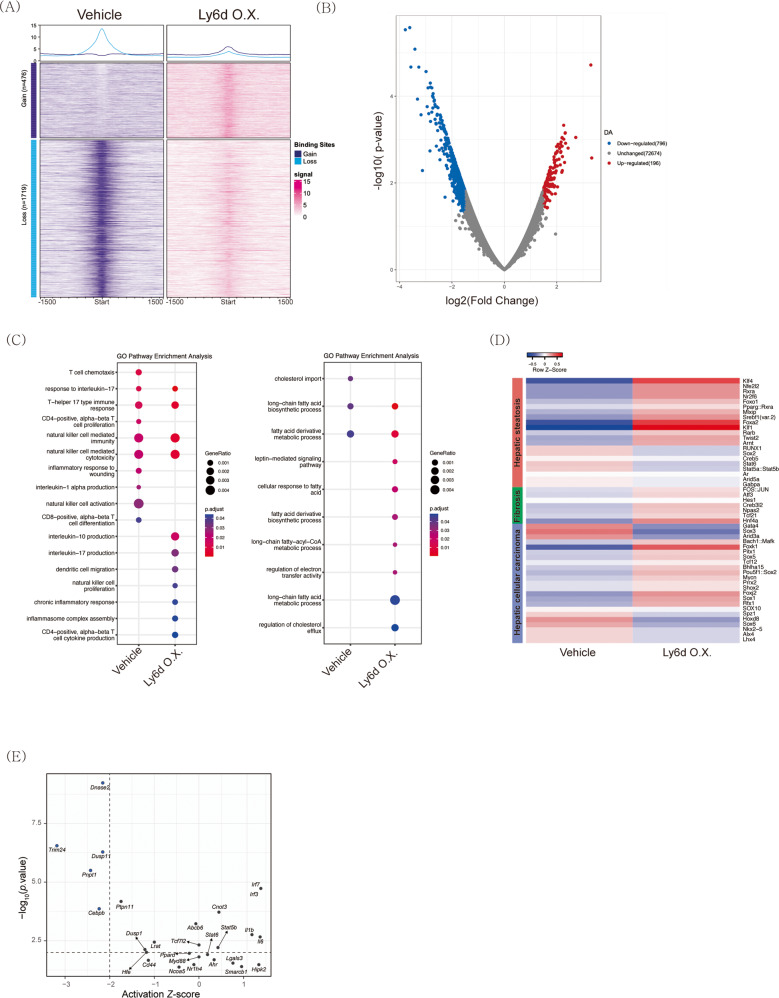
ATAC sequencing led to the identification of a subset of *Ly6d*-responsive genes. **A** Average plots (top) and heatmaps (bottom) of DARs in *Ly6d*-overexpressing AML12 cells. Average plot showing global chromatin accessibility changes across center and surrounding regions ( ± 1.5 Kb) of gain and loss regions. The heatmap represents the average normalized ATAC sequencing counts for DARs. Each row represents one DAR. **B** Volcano plot of ATAC sequencing peaks in *Ly6d*-overexpressing AML12 cells. Significant peaks are shown in pink (*p* < 0.05). **C** Dot plot of immune-related (left) and metabolism-related (right) GO pathway enrichment. The sizes of the dots correspond to the percentage of DARs in the pathway, while the color gradations correspond to the adjusted *p* value for each GO pathway. **D** Heatmap of TF motif activities. The color gradient represents *chromVAR* TF motif bias-corrected deviations. **E** Upstream regulator analysis showing the *Z* score-inferred activation state and *p* value of the regulator. Horizontal and vertical lines represent the threshold of significant *p* value and activation *Z* score, respectively. DARs differentially accessible regions.

To explore the dynamics of *Ly6d*-induced chromatin accessibility in hepatocytes, we performed ATAC-seq using *Ly6d*-overexpressing AML12 cells and control cells. Figure 4A shows the altered chromatin accessibility following *Ly6d* overexpression. A total of 796 downregulated and 196 upregulated differentially accessible regions (DARs) were identified (Fig. 4B). To interpret the biological meaning of the ATAC-seq data, we performed enrichment analysis for DARs between *Ly6d-*overexpressing cells and control cells. Genes around the DAR were enriched in proinflammatory pathways and fatty acid metabolism (Fig. 4C).

Transcription factor (TF) motif enrichment analysis using *chromVAR*^30^ revealed that the DNA-binding motifs of TFs related to NAFLD development and progression were significantly associated with *Ly6d* expression. In *Ly6d*-overexpressing cells, we found enrichment of TF-binding motifs related to steatosis, including those of retinoid X receptor alpha (*Rxra*)^31^, MLX-interacting protein (*Mlxip*)^32^, and sterol-regulatory element-binding transcription factor 1 (*Srebf1*)^33^ (Fig. 4D and Supplementary Fig. 2c). TF-binding motifs related to fibrosis and TF-binding motifs related to cancer development also showed positive enrichment (Fig. 4D). TF-binding motifs for cytochrome C oxidase subunit 5 A (*Cox5a*) showed negative enrichment, and its mRNA expression was also lower in *Ly6d*-overexpressing cells than in control cells (Supplementary Table 2).

To predict upstream factors associated with *Ly6d* overexpression, we performed upstream regulator analysis using IPA^®^^34^. Figure 4E shows the predicted upstream genes associated with *Ly6d* expression. Inhibition of the tripartite motif-containing 24 protein (*Trim24*), which has been reported as a suppressor of lipid accumulation and cancer development in the liver^35,36^, was most significantly associated with *Ly6d* expression.

### Suppression of *Ly6d* reduces lipid accumulation in mouse hepatocytes

**Fig. 5:**
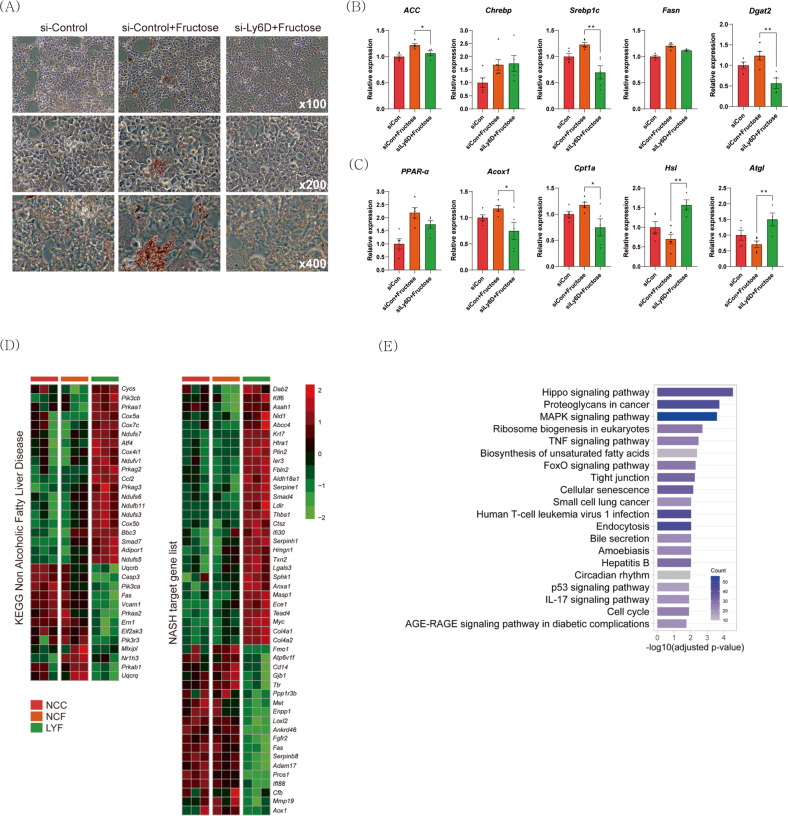
*Ly6d* inhibition suppresses lipid accumulation in hepatocytes. AML12 cells were transfected with siRNA, followed by incubation with 69.94 mM fructose for 24 h. si-Control control (NCC): scrambled siRNA-treated AML12 cells; si-Control+Fructose (NCF): scrambled siRNA-treated AML12 cells incubated with fructose; si-*Ly6D*: *Ly6D* knockdown AML12 cells; si-*Ly6D*+Fructose (LYF): *Ly6D* knockdown AML12 cells incubated with fructose. **A** Representative images of AML12 cells stained with Oil Red O. **B**, **C** qRT‒PCR analysis of hepatic gene expression related to lipogenesis **B** and lipolysis **C** (*n* = 6 in each group). **D** Heatmap visualizing the expression levels of genes related to NAFLD and NASH. **E** Bar chart summarizing the enriched KEGG pathways for DEGs between LYF and NCF. siRNA, short interfering RNA. The relative expression shown in **B** is presented as the mean ± SED, and the significance levels are *p < 0.05, **p < 0.01, and ***p < 0.001.

To determine whether *Ly6d* inhibition could suppress hepatic steatosis in vitro, we compared lipid accumulation in AML12 cells treated with *Ly6d*-specific short interfering RNA (KD cells) and those treated with nontargeting short interfering RNA (control cells). As expected, lipid accumulation was significantly reduced in KD cells compared to control cells (Fig. 5A and Supplementary Fig. 3a). As shown in Fig. 5B, C, the mRNA expression levels of lipogenic genes were downregulated, while those of lipolytic genes were upregulated in KD cells compared to control cells. To investigate the metabolic functions of *Ly6d* in hepatocytes more closely, we performed RNA-seq and identified DEGs between KD and control cells (Supplementary Fig. 3b). Compared to both untreated control cells and fructose-treated control cells, fructose-treated KD cells showed distinct gene expression patterns related to NAFLD and NASH (Fig. 5D). Steatosis-related genes, including *Mlxipl* and nuclear receptor subfamily 1 group H member 3 (*Nr1h3*)^37^, were suppressed, while mitochondrial respiratory chain genes were increased in fructose-treated KD cells compared to those in other cells. “Hippo signaling pathway”, “proteoglycans in cancer”, and “MAPK signaling pathways” were the significantly enriched KEGG pathways (Fig. 5E).

### Suppression of *Ly6d* ameliorates diet-induced hepatic steatosis in mice

**Fig. 6:**
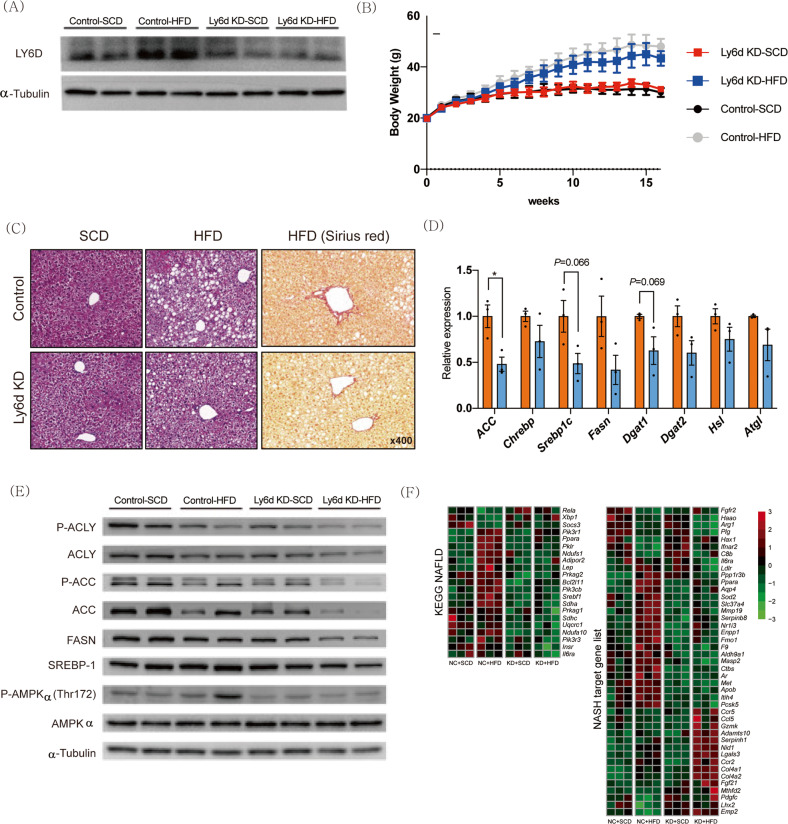
*Ly6d* inhibition prevents hepatic steatosis in HFD-fed mice. Mice were intravenously injected with AAV-scrambled shRNA (control) or AAV-sh*Ly6d* (Ly6d-KD) (*n* = 5 in each group). Control-SCD: SCD-fed control mice; Control-HFD: HFD-fed control mice; Ly6d KD-SCD: SCD-fed Ly6d KD mice; Ly6d-HFD: HFD-fed Ly6d KD mice. **A** Western blot analysis of the relative Ly6d protein levels in liver tissues. **B** Body weight changes in mice from 8 weeks to 24 weeks. **C** Images of H&E and Picrosirius Red staining of livers from control and Ly6d KD mice. **D** qRT‒PCR analysis of the mRNA expression levels of genes related to lipid metabolism in HFD-fed control (orange) and HFD-fed Ly6d KD (light blue) mice. **E** Western blot analysis of proteins related to lipogenesis. **F** Heatmap-based visualization of the expression levels of genes related to NAFLD and NASH. SCD, standard chow diet; HFD, high-fat diet. The average value displayed in **B** and **D** is presented as the mean ± SED. The significance level for the relative expression indicated in **D** is *p < 0.05.

To further explore the physiological role of Ly6d in NAFLD, we suppressed *Ly6d* expression in the liver by tail vein injection of a serotype 8 AAV expressing short hairpin RNA against *Ly6d*. After 16 weeks of HFD feeding, Western blot analysis showed that the expression levels of Ly6d protein were significantly lower in the livers of HFD-fed *Ly6d* KD mice than in those of HFD-fed control mice (Fig. 6A). During the study period, both control and HFD-fed *Ly6d* KD mice showed significantly greater body weight gain than SCD-fed mice (Fig. 6B). Histological analysis showed decreased hepatic lipid accumulation and reduced fibrosis in the livers of HFD-fed *Ly6d* KD mice compared with those of HFD-fed control mice (Fig. 6C). In addition, suppression of *Ly6d* expression in the liver also prevented HSD-induced hepatic steatosis (Supplementary Fig. 4a, b).

The hepatic expression levels of lipogenic genes were also lower in HFD-fed *Ly6d* KD mice than in HFD-fed control mice (Fig. 6D). Western blot analysis revealed a significant decrease in ACLY phosphorylation in HFD-fed *Ly6d* KD mice compared to HFD-fed control mice (Fig. 6E). Other core factors for de novo lipogenesis, such as acetyl-CoA carboxylase (Acc) and fatty acid synthase (Fasn), were lower in HFD-fed *Ly6d* KD mice than in HFD-fed control mice.

To investigate how *Ly6d* deficiency ameliorates diet-induced hepatic steatosis, we performed RNA-seq analysis of liver tissues from SCD-fed control mice, HFD-fed control mice, SCD-fed KD mice, and HFD-fed KD mice. DEG analysis revealed that lipogenic genes, including *Srebf1*, were downregulated, while antifibrotic genes, including fibroblast growth factor 21 (*Fgf21*), were upregulated in HFD-fed *Ly6d* KD mice compared to HFD-fed control mice (Fig. 6F)^38^. A volcano plot of DEGs between HFD-fed *Ly6d* KD mice and control mice revealed that genes related to hepatocyte-specific PPAR-α signaling were upregulated in the HFD-fed *Ly6d* KD mice (Supplementary Fig. 4c)^39^. Clustering and enrichment analysis showed that the enriched pathways in the cluster of the HFD-fed *Ly6d* KD mice included “regulation of lipid metabolic process”, “regulation of organelle transport along microtubule”, and “regulation of neurogenesis” (Supplementary Fig. 4d).

### ScRNA-seq reveals the potential role of Ly6d in nonparenchymal cells of the NAFLD mouse liver

**Fig. 7:**
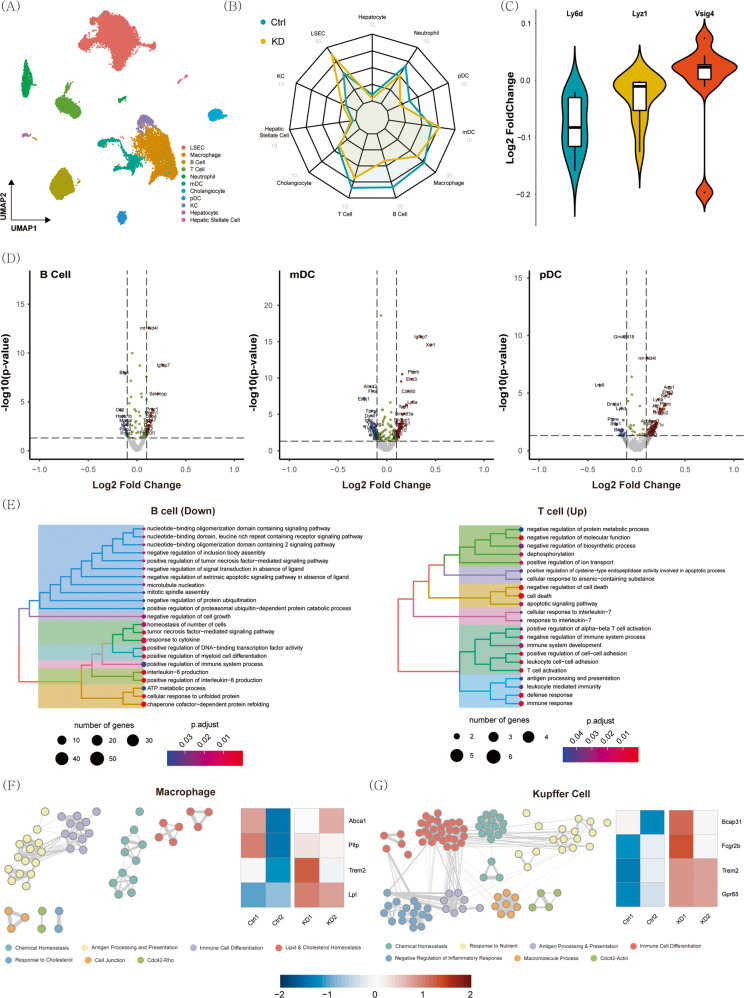
Single-cell RNA sequencing data from the livers of HFD-fed mice. The mice were intravenously injected with AAV-scrambled shRNA (Ctrl) or AAV-sh*Ly6d* (KD) and then fed a HFD. **A** UMAP plot for the integrated data. **B** Radar plot for the mean abundance of each cell type in the Ctrl and KD samples. **C** Violin plot for the log2-fold-change distribution of *Ly6d*, *Lyz1*, and *Vsig4* in each cell type. **D** Volcano plot for B cells, mDCs, and pDCs. DEGs were defined as genes with abs (log2-FC)≥0.1 and *p* < 0.05. **E** Hierarchical clustering of enriched GO terms for downregulated DEGs in B cells and upregulated DEGs in T cells. **F** Network representation of similarities between enriched GO terms for upregulated DEGs in macrophages and heatmap for genes playing a key role in lipid and cholesterol metabolism. **G** Network representation of similarities between enriched GO terms for upregulated DEGs in Kupffer cells and heatmap for genes playing a key role in inflammatory responses. HFD high-fat diet, mDCs myeloid dendritic cells, pDCs plasmacytoid dendritic cells.

To elucidate the role of Ly6d in nonparenchymal cells of the liver during NAFLD pathogenesis, we performed scRNA-seq on hepatocytes and nonparenchymal liver cells from HFD-fed control mice (CON, *n* = 2) and HFD-fed *Ly6d* KD mice (KD, *n* = 2). We identified 11 cell types after quality control, normalization, and manual annotation of the cell clusters using known signature genes (Fig. 7A and Supplementary Fig. 5a, b). The radiation plot in Fig. 7B shows the subtype fractions of each group. KD mice had higher fractions of liver sinusoidal endothelial and myeloid dendritic cells than CON mice. KD mice had smaller fractions of macrophages, B cells, T cells, and plasmacytoid dendritic cells than CON mice.

We then identified DEGs for each subtype (Supplementary Fig. 5C). Overall, *Ly6d* mRNA expression was lower in KD mice than in CON mice. Intriguingly, the overall expression of the proinflammatory marker lysozyme 1 (*Lyz1*)^40^ was decreased and that of the anti-inflammatory marker V-set and immunoglobulin domain-containing 4 (*Vsig4*)^41^ was increased in KD mice (Fig. 7C). We found that *Ly6d w*as highly expressed in dendritic cells and B cells among nonparenchymal cells (Supplementary Fig. 5d). We confirmed this finding in other liver transcriptome data (Supplementary Fig. 5e, GSE108097^42^, GSE176063^43^).

The DEGs in B cells and dendritic cells are shown in Fig. 7D. One of the highly significantly upregulated genes in both the DEGs of B cells and plasmacytoid dendritic cells was the mitochondrially encoded NADH dehydrogenase 4 L (mt-Nd4l). This gene encodes the core subunit of NADH dehydrogenase (complex 1 of the electron transport chain), which suppresses inflammation in immune cells^44,45^. Compared to those of HFD-fed CON mice, the B cells and myeloid dendritic cells of HFD-fed KD mice showed significant upregulation of the insulin-like growth factor-binding protein 7 (*Igfbp7*) gene (Fig. 7D), which has been shown to present anti-inflammatory effects on dendritic cells^46^. *Igfbp7* knockout mice displayed a proinflammatory status, with a high incidence of tumor development upon carcinogen treatment^46^.

We then conducted pathway analysis of the DEGs of each cell subtype (Fig. 7E and Supplementary Fig. 5f). In B cells, the most significant pathways related to proinflammation were negatively enriched. Macrophages and Kupffer cells in the liver play essential roles in NASH pathogenesis^47^. Thus, to further understand the role of these two critical immune cell types, we performed a network analysis. The strongly linked gene clusters in macrophages were associated with “antigen processing and presentation” and “immune cell differentiation” (Fig. 7F), while genes associated with “immune cell differentiation” and “negative control of inflammatory responses” formed highly connected clusters in Kupffer cells (Fig. 7G). In the cell‒cell communication analysis, macrophages showed increased interactions with other immune cells in HFD-fed KD mice (Supplementary Fig. 5g, h). The expression of the triggering receptor expressed on myeloid cells 2 (*Trem2*) gene, which has previously been shown to have protective effects against NAFLD progression in a mouse model^48,49^, was upregulated in HFD-fed KD mice compared to that in HFD-fed CON mice (Fig. 7F, G). These data suggest that *Ly6d* KD alleviates hepatic inflammation by suppressing proinflammatory signals of immune cells in the liver.

## Discussion

In the present study, we showed that both HFD and HSD increase hepatic steatosis in mice. Intriguingly, HSD-fed mice displayed more severe histological features in the liver than HFD-fed mice. In addition, HFD-fed mice had higher body weights and more severe insulin resistance but less hepatic steatosis than HSD-fed mice (Fig. 1D). Recent studies have provided supporting evidence for the role of high carbohydrate levels in the pathogenesis of NAFLD. High fructose beverage consumption has been associated with NAFLD in human studies^50^, and even high-carbohydrate with low-fat diets have been shown to induce hepatic steatosis and inflammation in animal models^51^. These results suggest that increased de novo lipogenesis via dietary carbohydrate intake is a significant factor in the development of NAFLD^52^. We also found that the expression of genes involved in ECM remodeling and immune responses gradually increased from the SCD to HFD to HSD groups (Fig. 1E). This could be attributed to the activation of hepatic stellate cells by carbohydrates. Recent studies suggest that high carbohydrate intake directly activates these cells^53–55^, which play a crucial role in the development and progression of liver cirrhosis^56^. An in vitro study by Piras et al. demonstrated that conditioned medium derived from hepatocytes treated with a combination of palmitate and fructose elicited a significantly greater degree of transcriptomic changes in hepatic stellate cells than conditioned medium from hepatocytes treated with palmitate alone^57^.

Upon comparing the transcriptome data between groups, we found that *Ly6d* expression was significantly associated with diet-induced NAFLD (Fig. 1E, F). Ly6d is a membrane-bound protein that attaches to the cell membrane as a glycosylphosphatidylinositol-anchored protein^58^. This protein functions as a surface marker of lymphocyte specificity^13^ and is associated with cancer progression^59^. Although several studies have found a link between *Ly6d* expression and hepatic steatosis^60,61^, the molecular mechanisms underlying Ly6d-mediated NAFLD development remain unknown. In this study, we demonstrated that *Ly6d* overexpression increased lipid accumulation, whereas *Ly6d* suppression decreased lipid accumulation in AML12 hepatocytes (Figs. 3B and 5A). In line with these results, *Ly6d* KD mice also displayed decreased hepatic steatosis in both the HFD- and HSD-fed groups (Fig. 6C and Supplementary Fig. 4b). Intriguingly, there was a significant increase in Acly phosphorylation in *Ly6d*-overexpressing hepatocytes (Fig. 3D). In contrast, there was a decrease in the protein expression of the main lipogenic enzyme in the livers of Ly6d KD mice (Fig. 6E). These findings suggested that Ly6d controls de novo lipogenesis by regulating the Acly-Acc axis.

TF motif analysis revealed that the TF-binding sites of key lipogenic TFs (including *Srebf1*, *Pparγ*, *Rxra*, and *Mlxip*) were significantly enriched upon Ly6d overexpression (Fig. 4E). Upstream regulator analysis proposed that Trim24 is an upstream negative regulator of Ly6d (Fig. 4E). Intriguingly, a specific *Trim24* knockout mouse displayed severe hepatic steatosis^35^. These results highlight Ly6d as a key mediator of lipogenesis in hepatocytes.

We performed scRNA-seq to assess the transcriptional changes in nonparenchymal cells and hepatocytes after *Ly6d* suppression in the livers of HFD-fed mice (Fig. 7). Compared to those in the livers of CON mice, B and T cells, which are essential immunological lymphocytes for the adaptive immune response, had lower relative abundance and positively enriched pathways involved in anti-inflammatory signaling in the livers of KD mice. These results suggest that *Ly6d* suppression regulates adaptive immunity in the liver, which is a key player in hepatic inflammation and fibrosis^62^. The relative abundance of immune cells, such as macrophages and neutrophils, was also lower in the livers of KD mice than in those of CON mice. Taken together, *Ly6d* suppression in the liver resulted in a reduced inflammatory status in the liver tissue of HFD-fed mice.

This study has limitations. To suppress *Ly6d* expression in the liver, we used AAV8 vector-mediated RNA interference in this study. Although the AAV8 serotype has a high affinity for hepatocytes^63^, nonparenchymal cell gene expression can be suppressed by this system. Thus, future studies could use hepatocyte-specific *Ly6d* knockout mice instead. In our single-cell RNA-seq data, the population of hepatocytes was low. This may be due to cellular damage to hepatocytes during the isolation process. As a result, the transcriptomic features of hepatocytes may not be accurately represented in our data. To overcome this limitation, further studies utilizing single-nucleus RNA-seq may provide a more accurate representation of the transcriptomic characteristics of hepatocytes.

In conclusion, we identified a new therapeutic target for NAFLD treatment. Ly6d contributes to the pathogenesis of NAFLD by regulating various genetic and epigenetic modifications. This study provides new insights into the pathophysiology of NAFLD and novel therapeutic targets for NAFLD.

## Supplementary information

Supplementary materials

## Data Availability

The sequencing data are accessible via the Gene Expression Omnibus database (GSE108097, GSE176063).
