# Supplementary material for: LY6D is crucial for lipid accumulation and inflammation in nonalcoholic fatty liver disease
Source: Exp Mol Med. 2023 Jul 3;55(7):1479–91. doi: 10.1038/s12276-023-01033-w (PMC10394021; doi:10.1038/s12276-023-01033-w)
Supplement: Supplementary file 1 — Supplementary materials [file 12276_2023_1033_MOESM1_ESM.pdf]

LY6D is crucial for lipid accumulation and inflammation in nonalcoholic fatty liver disease

Jibeom Lee, Hyeonhui Kim, Yun-Won Kang, Yumin Kim, Moon-young Park, Ji-Hong Song, Yunju Jo, Tam Dao, Dongryeol Ryu, Junguee Lee, Chang-Myung Oh, and Sangkyu Park

Table of contents

Supplementary methods ……..2

Supplementary Figure 1 ……..8

Supplementary Figure 2 ……..10

Supplementary Figure 3 ……..12

Supplementary Figure 4 ……..14

Supplementary Figure 5 ……..16

Supplementary Table 1 ……..19

Supplementary Table 2 ……..21

References ……..23

In vitro knockdown and overexpression of _Ly6d_

AML12 cells (5×10^5 cells/ml) were seeded into 6-well plates and grown for 24 h. The cells were then transfected with a short interfering RNA against *Ly6d* (Dharmacon, Lafayette, CO, USA), using Lipofectamine™ RNAiMAX Transfection Reagent (13778150, Invitrogen, Waltham, MA, USA), to knockdown *Ly6d in vitro*, followed by culture in Opti-MEM™ media (Thermo Fisher Scientific, Walthan, MA, USA) according to the manufacturer's instructions. After 6 h, the Opti-MEM™ medium was replaced with complete DMEM/F12 media containing fetal bovine serum, dexamethasone, and insulin transferrin solution, in addition to either 34.98 μM fructose or vehicle. After the samples were treated with fructose for 18 h, further experiments were performed.

Oil Red O staining

AML12 cells were seeded into 6-well plates, at a density of 1×10^6 cells/well. Ly6d knockdown cells were grown for 48 hours at 37°C in complete medium with or without 34.98 M fructose. To prepare the cells for Oil Red O staining, they were subjected to 10% formalin for 2 d, at 4°C, and subsequently allowed to incubate for 15 min at room temperature, before treatment with Oil Red O staining solution. The fixed cells were washed with ice-cold PBS at three times. To increase solution penetration, we treated the cells with 60% isopropanol for 5 min. Fresh 60% Oil Red O staining was filtered right after 2-isopropanol mixing, and the cells were stained with it for 30 min, at 25°C, on a shaking plate, following which they were washed three times with PBS, and then imaged.

Genotype-Tissue Expression (GTEx) data analysis

Correlations between hepatic *Ly6d* and all available phenotypes were calculated by applying

Spearman’s rank correlation, since the recorded phenotypes contained non-parametric data[1]. The R package *ggpubr* function was applied for the calculation, and the results were visualized using *ggplot2*. All BXD phenotypes are available on the GeneNetwork website (https://www.genenetwork.org/).

ATAC-seq and data analysis

Single-cell suspensions of AML12 were prepared using trypsin-EDTA, following which the cells were counted using a LUNA-FL™ Automated Fluorescence Cell Counter (Logos Biosystems, Anyang-si, Gyeonggi-do, South Korea). After cell lysis, the nuclear concentration was measured using a SOL COUNT automated cell counter (SOL Inc., Seoul, South Korea), and the morphology of the nuclei was examined. After lysis, the transposition reaction continued. Nuclei (50,000 cells) were resuspended in transposition reaction mix and incubated for 30 min at 37°C. Immediately following the transposition process, the sample was purified using a Qiagen MinElute® PCR Purification Kit. Transposed DNA fragments were amplified using a Nextera DNA Flex Kit ((Illumina, Inc., San Diego, CA, USA). A qPCR side reaction was performed with an appropriate number of cycles, to reduce GC and size bias. The amplified library was purified and quantified using the Quantification Protocol Guide (Kapa Biosystems, Wilmington, MA, USA), with the quality confirmed using a Bioanalyzer (Agilent Technologies, Santa Clara, CA, USA).

Libraries were sequenced using the HiSeq platform (Illumina). The experiments were performed in duplicate. Peaks were called with aligned sequence data using a model-based analysis of ATAC-seq (MACS2 version 2.1.1)[2]. Among the called peaks, those overlapping with the ENCODE blacklisted regions were removed. The ChIPseeker package (version 1.20.0)[3] was used to identify nearby genes and transcripts from peaks obtained using MACS2.

A heatmap of the normalized signal intensity across genomic regions was generated using DeepTools[4].

Upstream regulator analysis

Upstream regulator analysis was performed using IPA®, for DEGs from the denoted groups[5]. The *p*-value was calculated using Fisher's exact test, with a significance threshold of 0.05 and an activation *Z*-score threshold of ±2.

Pre-processing for scRNA-seq

The liver tissue was extracted and divided into four pieces, following which the left lobe was stored in fresh DMEM/F12 fluid for an hour. Using pathogen-free scissors, the stored tissues were cut into equal pieces and divided into individual cells. The number of cells were in the range of 0.72×10^5 to 1.88×10^6. The library used for scRNA-seq was BD WTA (BD Biosciences, San Jose, CA, USA) with 20,000 reads per cell. In the Q30 of FastQC, the quality checking rate was approximately 92% for all samples.

ScRNA-seq

The mitochondrial genome transcript ratio was calculated using the 'PercentageFeatureSet' function. Genes expressed by fewer than three cells were filtered out. Cells with a mitochondrial gene ratio >50% were excluded. Finally, the 'miQC' algorithm[6] was applied to each of the four samples independently, using the linear-type mixture model with a posterior cut-off of 0.75. The normalized data were obtained using the 'NormalizeData' function from the count matrix, while cell cycle scores for the S and G2M phases were estimated using the 'CellCycleScoring' function. We integrated the scRNA-seq data following the procedure suggested by Stuart et al.[7]. Variable features were identified by applying the 'NormalizeData'

and ‘FindVariableFeatures’ functions to the count matrix of each sample independently, from which 2000 integration features were selected using the ‘SelectIntegrationFeatures’ function. Anchors for the integration were identified using the ‘FindIntegrationAnchors’ function with 30 dimensions, to specify the neighbor search space. Finally, integration was performed using the ‘IntegrateData’ function.

For dimension reduction and analysis following that, the integrated data was scaled using the ‘ScaleData’ function, after regressing out mitochondrial gene ratio, unique molecular identifier (UMI) counts, S and G2M phase scores. Fifty principal components were calculated using the ‘RunPCA’ function. The dimension of the principal components was determined as the number of eigenvalues statistically larger than the maximum of those from the permuted matrix obtained by randomly shuffling the scaled expression values for each gene.

For visualization of the integrated data, Uniform Manifold Approximation and Projection (UMAP) was obtained from the principal components space, using the function ‘RunUMAP’. The integrated data were clustered by applying the ‘FindNeighbors’ and ‘FindClusters’ functions in series to the principal components space. A resolution value of 0.4 was used to assign the cell type to each cluster using the expression of the marker gene set. In the radar plot, the ‘radarchart’ function from the ‘fmsb’ package was used. The marker gene set for each cell type was identified using the ‘FindAllMarkers’ function, while the ‘pheatmap’ package (version 1.0.12) was used to generate the heatmap of Top 10 marker genes of each cell type, ordered by the average log2 fold-change (log2-FC) value.

Genes with a *p*-value ≤0.05, absolute log2-FC value ≥0.1, and an expressed cell ratio ≥0.1 in both the groups were identified as the DEGs. The ‘EnhancedVolcano’ package was used to generate the volcano plots. Gene Ontology (GO) term analysis[8] was conducted using the

‘enricher’ function from the ‘clusterProfiler’ package (version 4.4.4)[9], for upregulated and downregulated genes separately. For GO term analysis, we used the Biological Process ontology terms, using all genes in the count matrix as background genes and a minimum gene set size of 5. Enriched GO terms with Benjamini–Hochberg adjusted p<0.05 were used for the following analysis.

Tree plots were used to visualize the hierarchy between the enriched GO terms. Semantic similarities between GO terms were measured using the ‘mgoSim’ function from the ‘GOSemSim’ package (version 2.22.0), from which GO terms were hierarchically clustered into 20 groups. Following that, for each of the 20 clusters, we selected the most significant GO terms with the minimum adjusted *p*-value, *q*-value, and maximum gene counts. If more than one GO term could have not been prioritized using all these three statistics, we used them all to draw tree plots, using the function ‘treeplot’ from the ‘enrichplot’ package (version 1.16.2).

For the network representation of GO terms, the ‘pairwise_termsim’ function from the ‘enrichplot’ package was used to calculate the Jaccard similarity between GO terms and assign it to the associated edge. Edges with a similarity of less than the 90% quantile of all similarity values were eliminated. Finally, the network was imported into Cytoscape (version 3.9.2)[10], and clustered using the ‘GLay’ algorithms[11] for community clustering provided by the ‘clusterMaker2’ plugin[12].

Generation of loss-of-function Ly6d in mice model, using adeno-associated virus 8 (AAV8)

Seven-week-old C57BL6/J male mice were first habituated to a contingency of 25°C. We then performed *Ly6d* mRNA knockdown specifically in the mouse liver using an AAV8 virus [AAV8MP (VB900050-8230sxe)-C, Vector Builder (Chicago, IL, USA)] encoding a *Ly6d*-targeting short hairpin RNA, with a confluency of 1×10^{11} viral genomes in 40 µl. The virus

was injected into the tail-veins of the mice when they were 8 weeks of age, immediately after which they were fed two types of diet, SCD or HFD. The knockdown results were confirmed 8 weeks after the injection and diet.

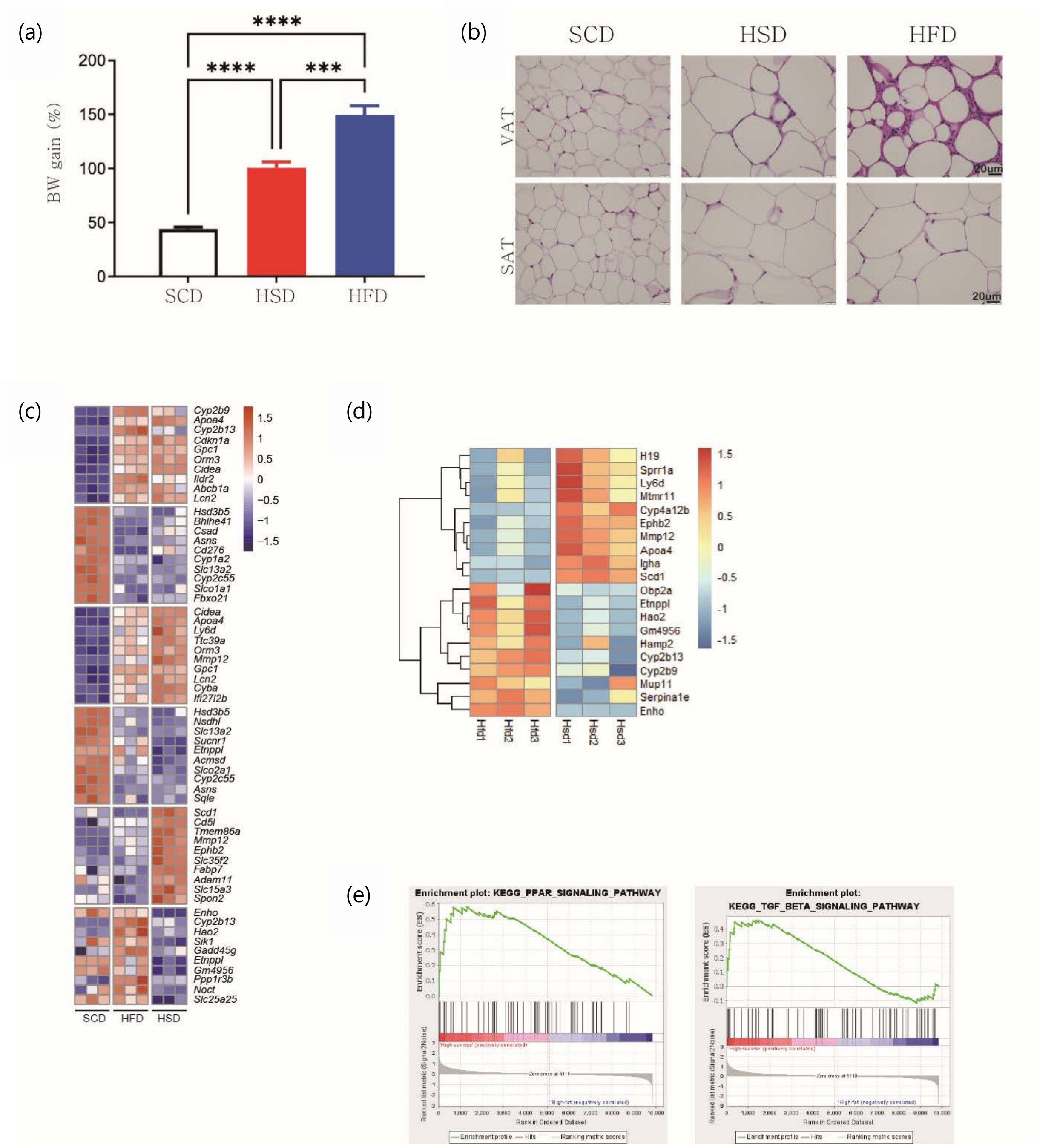

Supplementary Fig. 1. (a) Body weight changes of each group. Male mice were put on standard chow diet (SCD), high sucrose and high fat diet (HSD), and high fat diet (HFD). (b) Hematoxylin and eosin (H&E) of visceral adipose tissue (VAT) and subcutaneous adipose tissue (SAT). Scale bar=20μm. (c) Heatmap of significantly expressed genes by DEGs analysis between three groups (p-value < 0.05 and absolute value of log2-fold change (log2FC) > 1).

(d) Heatmap of highest 10 and the lowest 10 DEGs between mice with HSD and mice with HFD. The library aligned count value were calculated as DEseq2 and confirmed the highest 10 and the lowest 10 DEGs depending on log2FC. (e) Gene set enrichment analysis enrichment plots to PPAR-signaling pathway and TGF beta signaling pathway.

(a)

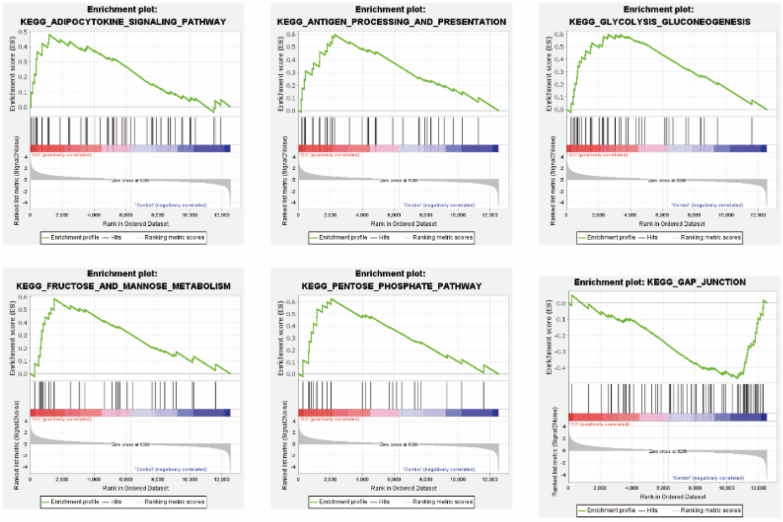

Enrichment plot: KEGG_ADIPOCYTOKINE_SIGNALING_PATHWAY
Enrichment plot: KEGG_ANTIGEN_PROCESSING_AND_PRESENTATION
Enrichment plot: KEGG_GLYCOLYSIS_GLUCONEOGENESIS
Enrichment plot: KEGG_FRUCTOSE_AND_MANNOSE_METABOLISM
Enrichment plot: KEGG_PENTOSE_PHOSPHATE_PATHWAY
Enrichment plot: KEGG_GAP_JUNCTION

(b)

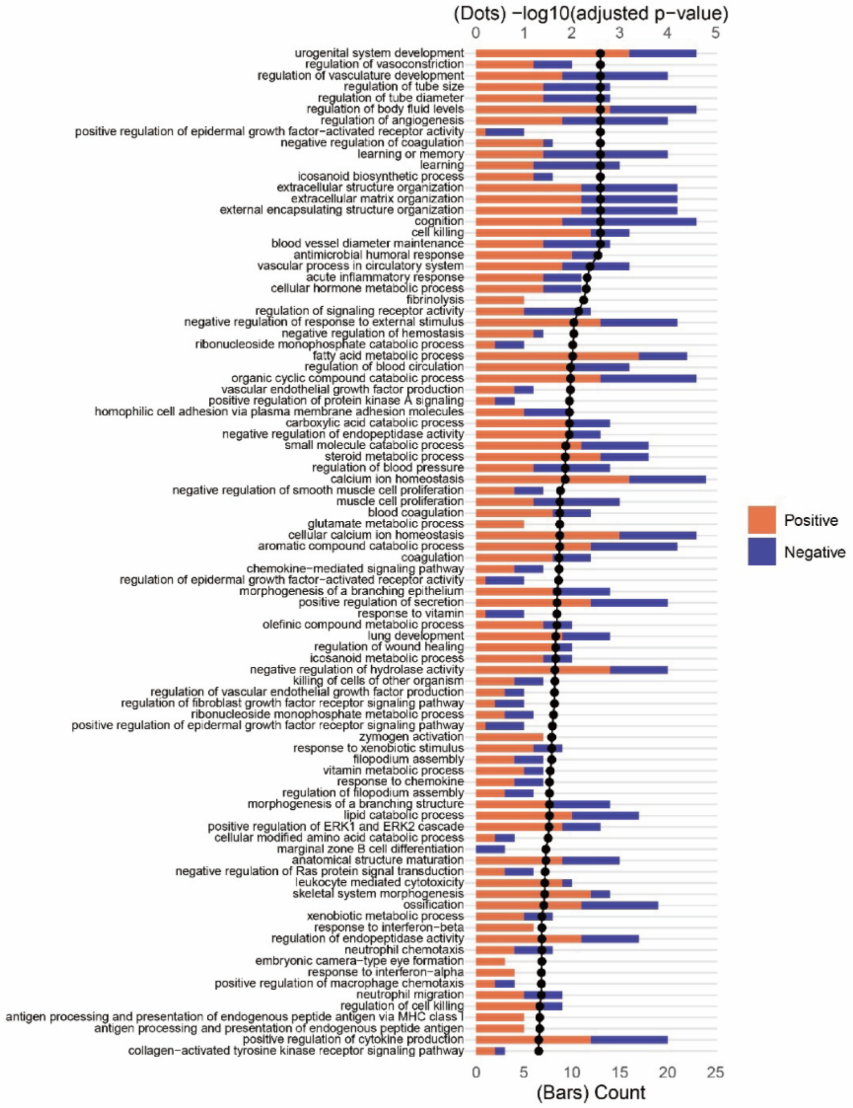

(Dots) −log10(adjusted p-value)
0
1
2
3
4
5
urogenital system development
regulation of vasoconstriction
regulation of vasculature development
regulation of tube size
regulation of tube diameter
regulation of body fluid levels
regulation of angiogenesis
positive regulation of epidermal growth factor−activated receptor activity
negative regulation of coagulation
learning or memory
learning
icosanoid biosynthetic process
extracellular structure organization
extracellular matrix organization
external encapsulating structure organization
cognition
cell killing
blood vessel diameter maintenance
antimicrobial humoral response
vascular process in circulatory system
acute inflammatory response
cellular hormone metabolic process
fibrinolysis
regulation of signaling receptor activity
negative regulation of response to external stimulus
negative regulation of hemostasis
ribonucleoside monophosphate catabolic process
fatty acid metabolic process
regulation of blood circulation
organic cyclic compound catabolic process
vascular endothelial growth factor production
positive regulation of protein kinase A signaling
homophilic cell adhesion via plasma membrane adhesion molecules
carboxylic acid catabolic process
negative regulation of endopeptidase activity
small molecule catabolic process
steroid metabolic process
regulation of blood pressure
calcium ion homeostasis
negative regulation of smooth muscle cell proliferation
muscle cell proliferation
blood coagulation
glutamate metabolic process
cellular calcium ion homeostasis
aromatic compound catabolic process
coagulation
chemokine−mediated signaling pathway
regulation of epidermal growth factor−activated receptor activity
morphogenesis of a branching epithelium
positive regulation of secretion
response to vitamin
olefinic compound metabolic process
lung development
regulation of wound healing
icosanoid metabolic process
negative regulation of hydrolase activity
killing of cells of other organism
regulation of vascular endothelial growth factor production
regulation of fibroblast growth factor receptor signaling pathway
ribonucleoside monophosphate metabolic process
positive regulation of epidermal growth factor receptor signaling pathway
zymogen activation
response to xenobiotic stimulus
filopodium assembly
vitamin metabolic process
response to chemokine
regulation of filopodium assembly
morphogenesis of a branching structure
lipid catabolic process
positive regulation of ERK1 and ERK2 cascade
cellular modified amino acid catabolic process
marginal zone B cell differentiation
anatomical structure maturation
negative regulation of Ras protein signal transduction
leukocyte mediated cytotoxicity
skeletal system morphogenesis
ossification
xenobiotic metabolic process
response to interferon−beta
regulation of endopeptidase activity
neutrophil chemotaxis
embryonic camera−type eye formation
response to interferon−alpha
positive regulation of macrophage chemotaxis
neutrophil migration
regulation of cell killing
antigen processing and presentation of endogenous peptide antigen via MHC class I
antigen processing and presentation of endogenous peptide antigen
positive regulation of cytokine production
collagen−activated tyrosine kinase receptor signaling pathway
0
5
10
15
20
25
(Bars) Count
Positive
Negative

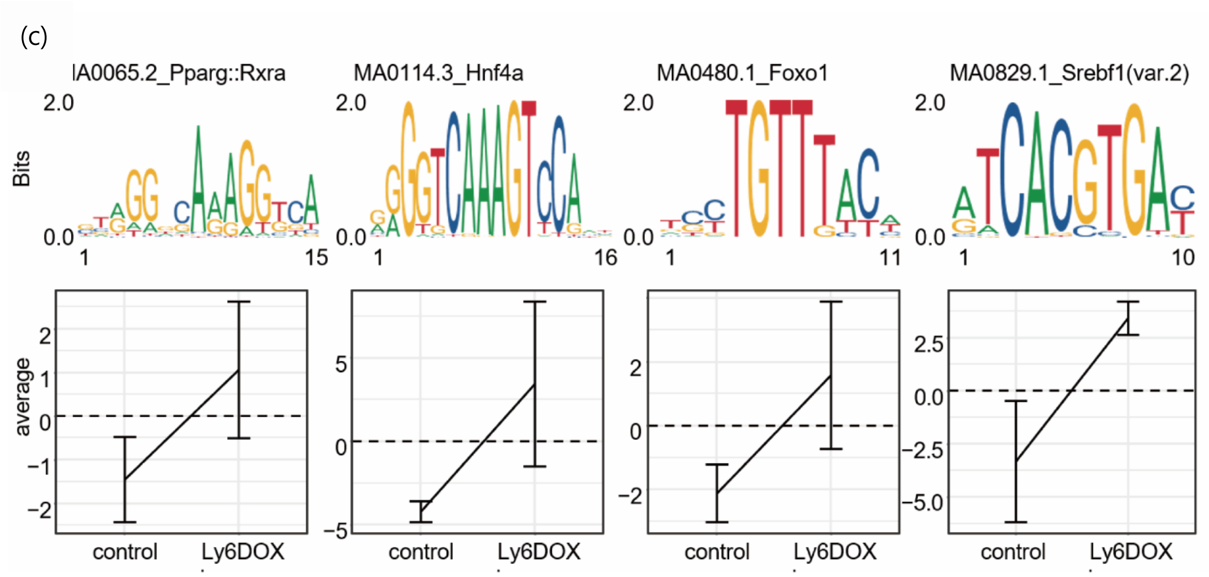

Supplementary Fig. 2. (a) Gene set enrichment analysis enrichment plots for differentially expressed genes between *Ly6d* overexpressed cells (O.X.) and control cells (Vehicle). (b) Bar chart of gene ontology enriched in biological process (adjusted p-value < 0.05 and |log2FC| >1). (c) Motif analysis results.

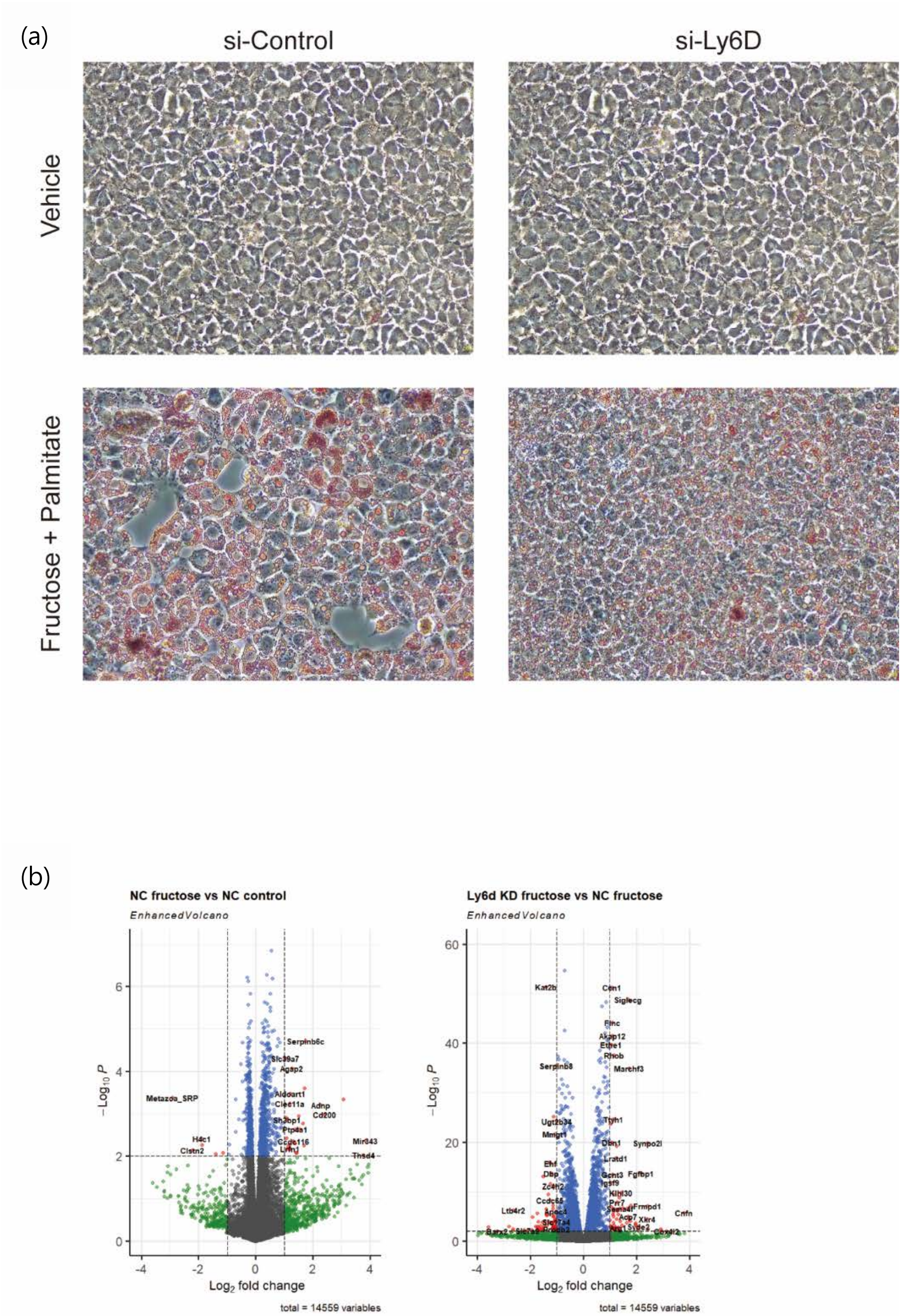

Supplementary Fig. 3. (a) Oil red O staining for lipid accumulation (x200 Magnification). AML12 cells were transfected with short interfering RNA (siRNA), then incubated with fructose (69.96 mM) + palmitate (1.5 mM) for 24 hours. si-Control: scrambled siRNA treated cells; si-*Ly6D*: *Ly6D* knockdown cells. (b) Volcano plots showing differentially expressed genes between groups. NC: cells transfected with si-Control; Ly6d KD: cells transfected with

si-*Ly6D*; NC fructose: NC incubated with fructose; NC control: nontreated NC; Ly6d KD fructose: Ly6d KD incubated with fructose.

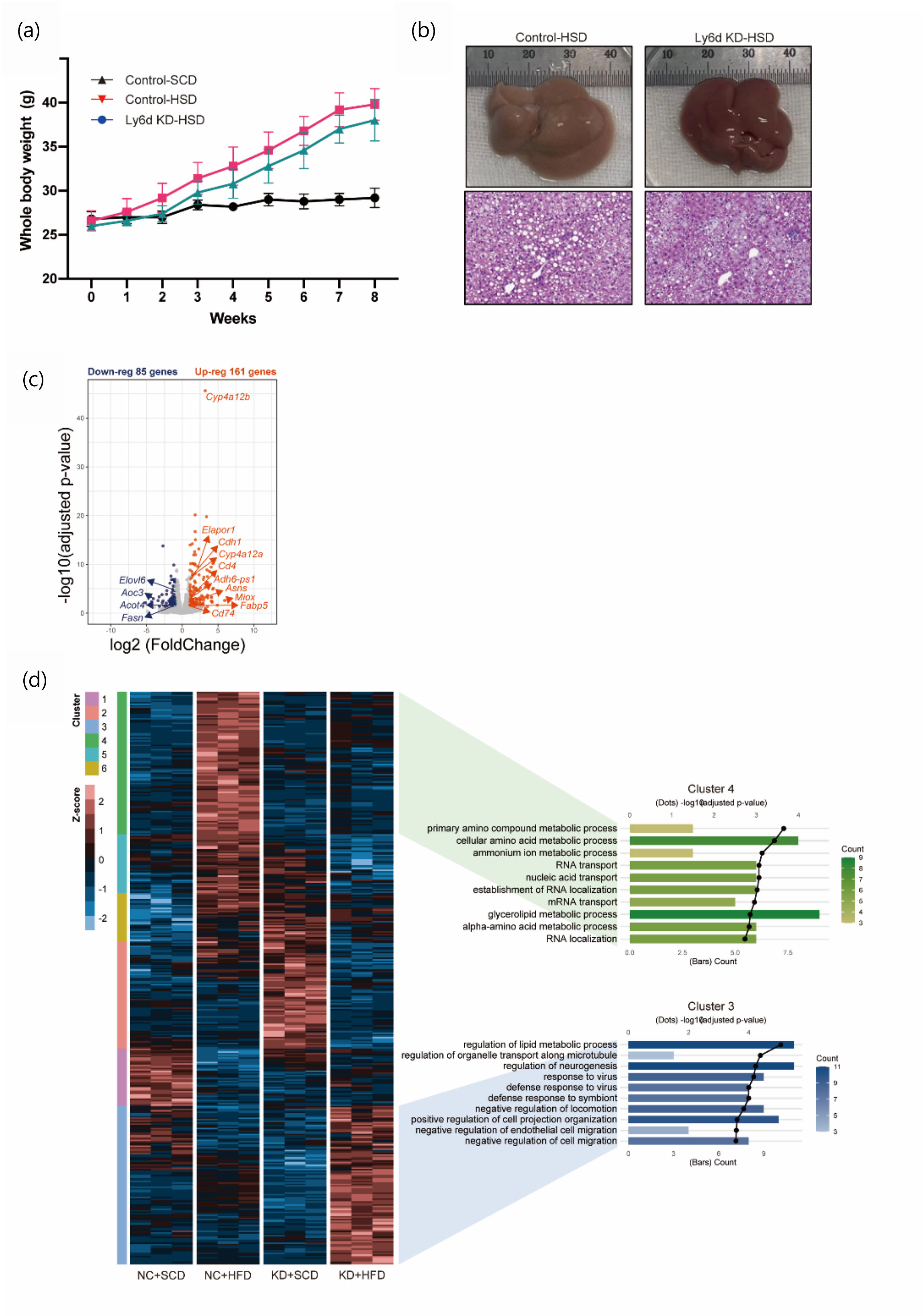
(a)
Control-SCD
Control-HSD
Ly6d KD-HSD
Whole body weight (g)
Weeks
(b)
Control-HSD
Ly6d KD-HSD
(c)
Down-reg 85 genes
Up-reg 161 genes
Cyp4a12b
Elapor1
Cdh1
Cyp4a12a
Cd4
Adh6-ps1
Asns
Miox
Fabp5
Cd74
Elovl6
Aoc3
Acot4
Fasn
-log10(adjusted p-value)
log2 (FoldChange)
(d)
Cluster
Z-score
NC+SCD
NC+HFD
KD+SCD
KD+HFD
Cluster 4
(Dots) -log10(adjusted p-value)
primary amino compound metabolic process
cellular amino acid metabolic process
ammonium ion metabolic process
RNA transport
nucleic acid transport
establishment of RNA localization
mRNA transport
glycerolipid metabolic process
alpha-amino acid metabolic process
RNA localization
(Bars) Count
Count
Cluster 3
(Dots) -log10(adjusted p-value)
regulation of lipid metabolic process
regulation of organelle transport along microtubule
regulation of neurogenesis
response to virus
defense response to virus
defense response to symbiont
negative regulation of locomotion
positive regulation of cell projection organization
negative regulation of endothelial cell migration
negative regulation of cell migration
(Bars) Count
Count

Supplementary Fig. 4. (a) Body weight change of mice fed a standard chow diet (SCD), in high sucrose high fat diet (HSD). Mice were intravenously injected with AAV-scrambled shRNA (Control) or AAV-sh*Ly6d* (Ly6d KD). (b) Macroscopic view and H&E staining images of livers from Control and Ly6d KD mice fed HSD. (c) Volcano plot showing differentially expressed genes between the livers of control mice with high fat diet (NC+HFD) and Ly6d KD mice with high fat diet (KD+HFD). Cut off: adjusted p-value<0.05 and |log2FC| >1. (d) Heatmap visualizing hierarchical clustering. A two-way ANOVA was used to produce the count table after two factors—diets and *Ly6d* expression levels.

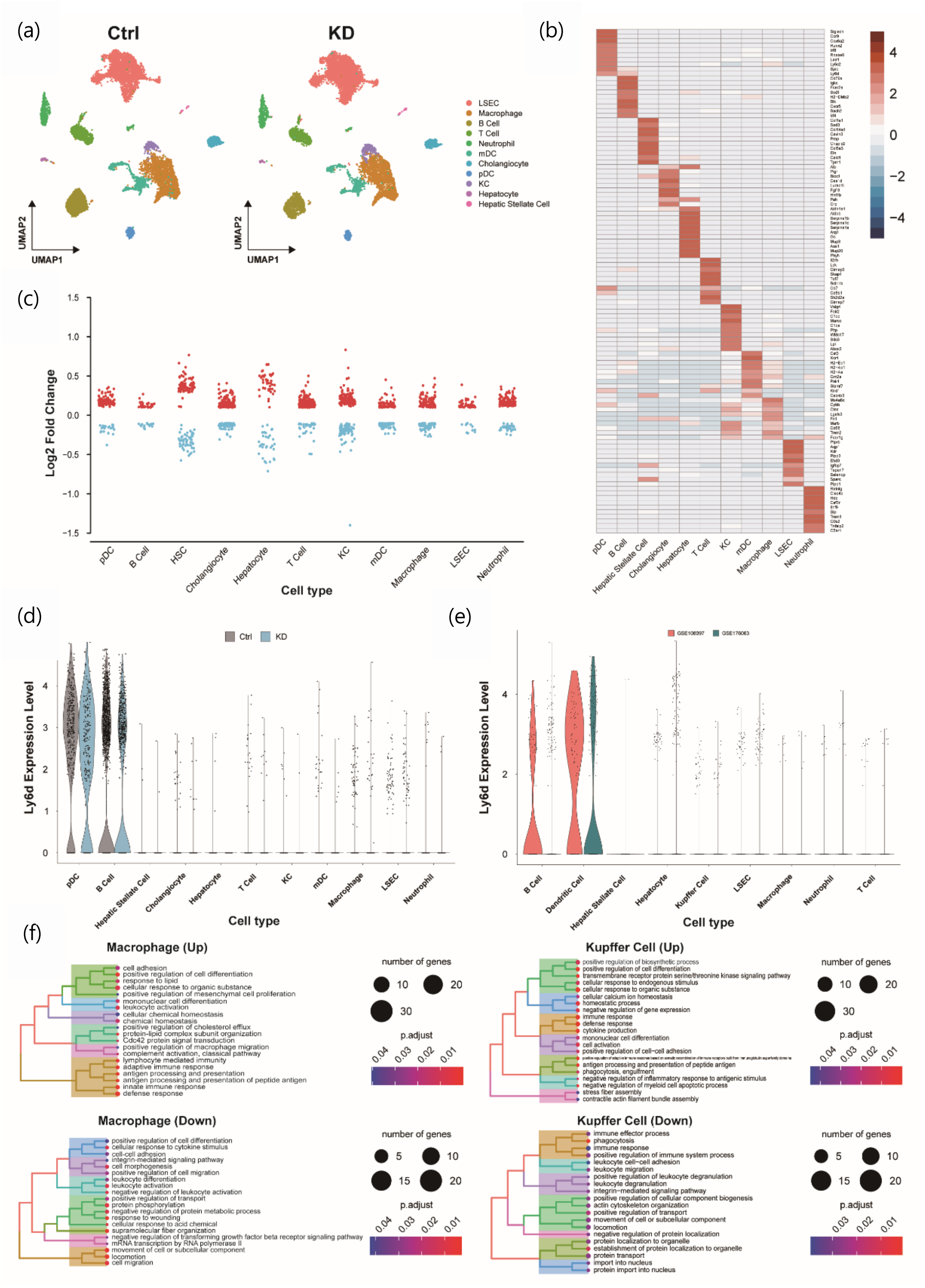

(a)
Ctrl
KD
LSEC
Macrophage
B Cell
T Cell
Neutrophil
mDC
Cholangiocyte
pDC
KC
Hepatocyte
Hepatic Stellate Cell
UMAP2
UMAP1
(b)
4
2
0
−2
−4
pDC
B Cell
Hepatic Stellate Cell
Cholangiocyte
Hepatocyte
T Cell
KC
mDC
Macrophage
LSEC
Neutrophil
(c)
Log2 Fold Change
1.5
1.0
0.5
0.0
−0.5
−1.0
−1.5
pDC
B Cell
HSC
Cholangiocyte
Hepatocyte
T Cell
KC
mDC
Macrophage
LSEC
Neutrophil
Cell type
(d)
Ctrl
KD
Ly6d Expression Level
pDC
B Cell
Hepatic Stellate Cell
Cholangiocyte
Hepatocyte
T Cell
KC
mDC
Macrophage
LSEC
Neutrophil
Cell type
(e)
GSE108097
GSE176063
Ly6d Expression Level
B Cell
Dendritic Cell
Hepatic Stellate Cell
Hepatocyte
Kupffer Cell
LSEC
Macrophage
Neutrophil
T Cell
Cell type
(f)
Macrophage (Up)
cell adhesion
positive regulation of cell differentiation
response to lipid
cellular response to organic substance
positive regulation of mesenchymal cell proliferation
mononuclear cell differentiation
leukocyte activation
cellular chemical homeostasis
chemical homeostasis
positive regulation of cholesterol efflux
protein-lipid complex subunit organization
Cdc42 protein signal transduction
positive regulation of macrophage migration
complement activation, classical pathway
lymphocyte mediated immunity
adaptive immune response
antigen processing and presentation
antigen processing and presentation of peptide antigen
innate immune response
defense response
number of genes
p.adjust
Kupffer Cell (Up)
positive regulation of biosynthetic process
positive regulation of cell differentiation
transmembrane receptor protein serine/threonine kinase signaling pathway
cellular response to endogenous stimulus
cellular response to organic substance
cellular calcium ion homeostasis
homeostatic process
negative regulation of gene expression
immune response
defense response
cytokine production
mononuclear cell differentiation
cell activation
positive regulation of cell−cell adhesion
antigen processing and presentation of peptide antigen
phagocytosis, engulfment
negative regulation of inflammatory response to antigenic stimulus
negative regulation of myeloid cell apoptotic process
stress fiber assembly
contractile actin filament bundle assembly
Macrophage (Down)
positive regulation of cell differentiation
cellular response to cytokine stimulus
cell-cell adhesion
integrin-mediated signaling pathway
cell morphogenesis
positive regulation of cell migration
leukocyte differentiation
leukocyte activation
negative regulation of leukocyte activation
positive regulation of transport
protein phosphorylation
negative regulation of protein metabolic process
response to wounding
cellular response to acid chemical
supramolecular fiber organization
negative regulation of transforming growth factor beta receptor signaling pathway
mRNA transcription by RNA polymerase II
movement of cell or subcellular component
locomotion
cell migration
Kupffer Cell (Down)
immune effector process
phagocytosis
immune response
positive regulation of immune system process
leukocyte cell−cell adhesion
leukocyte migration
positive regulation of leukocyte degranulation
leukocyte degranulation
integrin−mediated signaling pathway
positive regulation of cellular component biogenesis
actin cytoskeleton organization
positive regulation of transport
movement of cell or subcellular component
locomotion
negative regulation of protein localization
protein localization to organelle
establishment of protein localization to organelle
protein transport
import into nucleus
protein import into nucleus

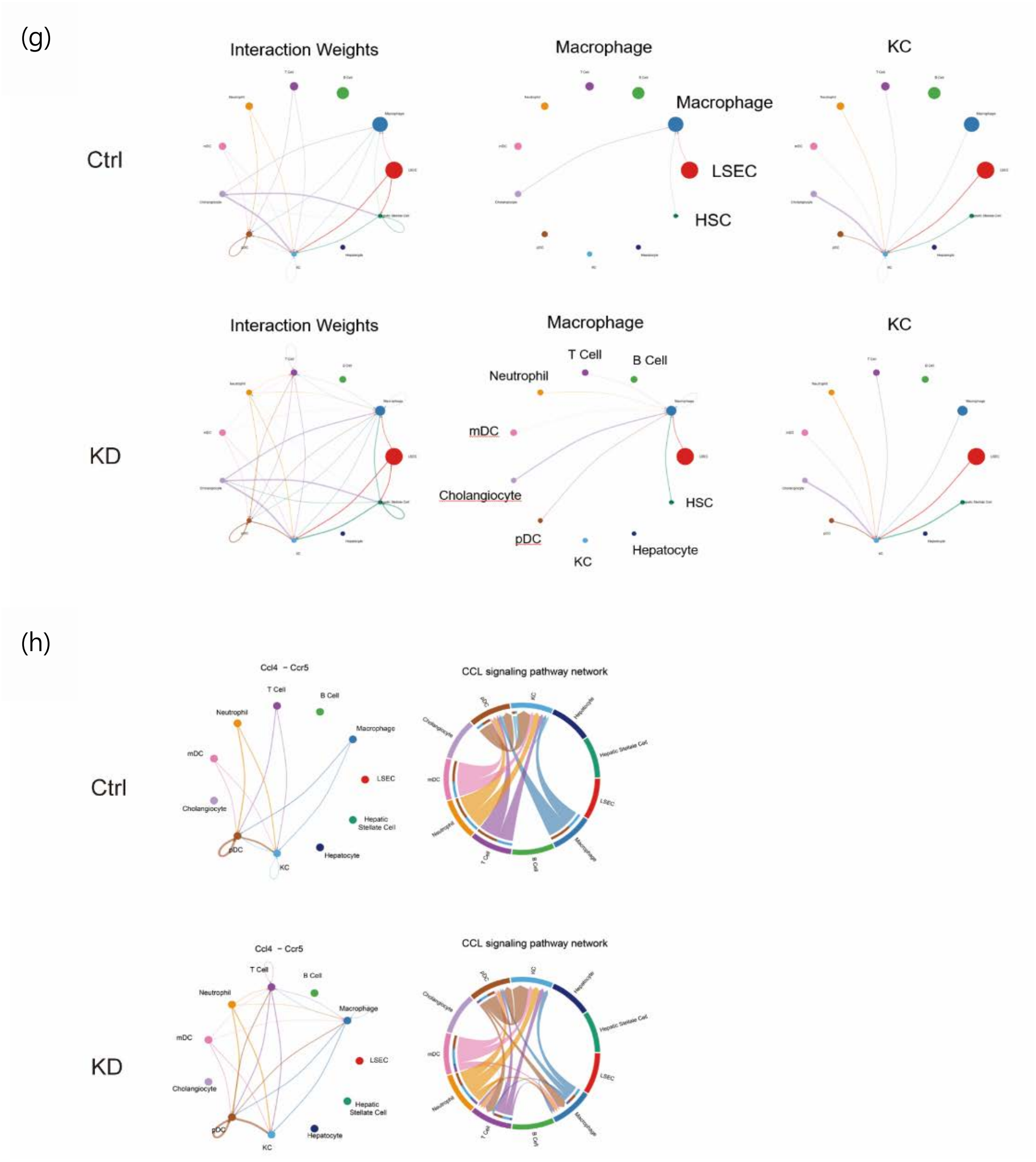

Supplementary Fig. 5. Mice were intravenously injected with AAV-scrambled shRNA (Ctrl) or AAV-sh*Ly6d* (KD), then fed a high fat diet. (a) UMAP plot for single cell sequencing data from livers of Ctrl and KD mice, each comprising two samples. (b) Heatmap for conserved marker genes in each cell type. (c) Log2 fold-change distribution of DEGs in each cell type. (d) Distribution of Ly6d expression level of each cell type in our dataset. (e) Distribution of Ly6d expression level of each cell type in two GEO datasets. (f) Hierarchical clustering of

enriched GO terms for up- and down- regulated DEGs in Macrophage and Kupffer Cell, respectively. (g, h). Cell-cell communication in the immune cells reconstructed with CellChat analysis.

Supplementary Table 1. Detailed DEGs list. The intersection in 3 groups of RNA sequencing DEGs results of diets induced NAFLD mouse model was detailed in the table. DEG: differentially expressed genes.

Gene	Description	Ref.
Apoa4	Apoliprotein (apo) A-IV	13
Axl	AXL Receptor Tyrosine Kinase	14
C8b	Subunits of the complement component 8 (C8) protein.	15
Ccl6	Chemokine 6	16
Ccr2	Chemokine (C-C Motif) Receptor 2	17
Cd44	CD44 molecule	18
Cd52	CD52 molecule	19
Cgref1	Cell Growth Regulator With EF-Hand Domain 1	20
Cidea	Activate apoptosis	21
Cidec	cell death-inducing DFFA-like effector c	22
Cntnap1	Contactin-Associated Protein 1	
Csad	Cysteine Sulfinic Acid Decarboxylase	23
Cyp2b9	Member of the cytochrome P450 superfamily of enzymes	24
Epop	Elongin BC And Polycomb Repressive Complex 2-Associated Protein	
Gpnmb	Glycoprotein Nmb	25
Ildr2	Immunoglobulin Like Domain Containing Receptor 2	26
Lgals3	Galectin family of carbohydrate binding proteins	27
Ly6d	Lymphocyte antigen family 6 d	20
Mmp12	Matrix Metalloproteinase 12	28
Tmem86a	Transmembrane Protein 86A	20
Tmsb4x	Thymosin, Beta 4, X Chromosome	29
Treh	Alpha-Trehalose Glucohydrolase	

Ttc39a	Tetratricopeptide Repeat Domain 39A	[30]

Supplementary Table 2. Comparison of ATAC sequencing and RNA sequencing data from *Ly6d* over-expression model. OX UP: upregulated in overexpressed cells compared with control cells. OX DOWN: downregulated in overexpressed cells compared with control cells.

Ensembl ID	Gene Symbol	Description	baseMean	log2FC	p-value	padj	RNA seq	ATAC seq
ENSMUSG00000034634	Ly6d	lymphocyte antigen 6 complex, locus D	75.3289	8.76862	5.99E-13	1.83E-11	UP	OX UP
ENSMUSG00000052776	Oas1a	2'-5' oligoadenylate synthetase 1A	36.53942	7.718777	6.47E-10	1.36E-08	UP	OX UP
ENSMUSG00000078597	Cyp4a12b	cytochrome P450, family 4, subfamily a, polypeptide 12B	12.76823	7.17289	1.59E-07	2.28E-06	UP	OX UP
ENSMUSG00000000385	Tmprss2	transmembrane protease, serine 2	1101.724	0.884318	1.22E-22	9.42E-21	UP	OX UP
ENSMUSG00000000594	Gm2a	GM2 ganglioside activator protein	1472.055	0.848105	1.37E-28	1.43E-26	UP	OX UP
ENSMUSG00000000693	Loxl3	lysyl oxidase-like 3	120.4025	0.766192	0.001641	0.009133	UP	OX UP
ENSMUSG00000000628	Hk2	hexokinase 2	284.6934	0.697483	3.33E-05	0.000294	UP	OX UP
ENSMUSG00000000126	Wnt9a	wingless-type MMTV integration site family, member 9A	211.6136	0.688912	0.000406	0.002689	UP	OX UP
ENSMUSG00000000326	Comt	catechol-O-methyltransferase	5447.448	0.385477	3.70E-10	8.09E-09	UP	OX UP
ENSMUSG00000000275	Trim25	tripartite motif-containing 25	3609.596	0.237224	4.09E-05	0.000353	UP	OX UP
ENSMUSG00000000711	Rab5b	RAB5B, member RAS oncogene family	3214.434	0.229042	0.000103	0.000796	UP	OX UP

ENSMUSG000 00000708	Kat2b	K(lysine) acetyltransferase 2B	1626.841	0.213504	0.002673	0.013752	UP	OX UP
ENSMUSG000 00000194	Gpr107	G protein-coupled receptor 107	2296.05	0.206964	0.001011	0.005992	UP	OX UP
ENSMUSG000 00000605	Clcn4	chloride channel, voltage-sensitive 4	2152.442	-0.23574	0.005209	0.023983	DOW N	OX UP
ENSMUSG000 00000384	Tbrg4	transforming growth factor beta regulated gene 4	2660.731	-0.24605	0.000418	0.00276	DOW N	OX UP
ENSMUSG000 00000088	Cox5a	cytochrome c oxidase subunit 5A	1152.301	-0.26357	0.001784	0.009802	DOW N	OX DOW N
ENSMUSG000 00000078	Klf6	Kruppel-like factor 6	5123.593	-0.33511	1.59E-10	3.67E-09	DOW N	OX UP
ENSMUSG000 00000339	Rtca	RNA 3'-terminal phosphate cyclase	1127.13	-0.36249	2.74E-05	0.000247	DOW N	OX UP
ENSMUSG000 00000561	Wdr77	WD repeat domain 77	1050.1	-0.44552	1.45E-05	0.000138	DOW N	OX UP
ENSMUSG000 00000555	Itga5	integrin alpha 5 (fibronectin receptor alpha)	2222.999	-0.48517	1.27E-11	3.30E-10	DOW N	OX UP
ENSMUSG000 00000184	Ccnd2	cyclin D2	2940.455	-0.54855	2.73E-20	1.74E-18	DOW N	OX UP
ENSMUSG000 00000296	Tpd52l1	tumor protein D52-like 1	846.8392	-0.83842	1.20E-13	3.99E-12	DOW N	OX UP

References

1 Chung, H. *et al.* Artificial-intelligence-driven discovery of prognostic biomarker for sarcopenia. *J. Cachexia Sarcopenia Muscle* **12**, 2220-2230 (2021).

2 Zhang, Y. *et al.* Model-based analysis of ChIP-Seq (MACS). *Genome Biol.* **9**, 1-9 (2008).

3 Yu, G., Wang, L.-G. & He, Q.-Y. J. B. ChIPseeker: an R/Bioconductor package for ChIP peak annotation, comparison and visualization. *Bioinformatics* **31**, 2382-2383 (2015).

4 Ramírez, F. *et al.* deepTools2: a next generation web server for deep-sequencing data analysis. *Nucleic Acids Res.* **44**, W160-W165 (2016).

5 Krämer, A., Green, J., Pollard, J. & Tugendreich, S. Causal analysis approaches in Ingenuity Pathway Analysis. *Bioinformatics* **30**, 523-530 (2014).

6 Hippen, A. A. *et al.* miQC: An adaptive probabilistic framework for quality control of single-cell RNA-sequencing data. *PLoS Comput. Biol.* **17**, e1009290 (2021).

7 Stuart, T. *et al.* Comprehensive integration of single-cell data. *Cell* **177**, 1888-1902. e1821 (2019).

8 Yu, G. *et al.* GOSemSim: an R package for measuring semantic similarity among GO terms and gene products. *Bioinformatics* **26**, 976-978 (2010).

9 Wu, T. *et al.* clusterProfiler 4.0: A universal enrichment tool for interpreting omics data. *Innovation (Camb.)* **2**, 100141 (2021).

10 Shannon, P. *et al.* Cytoscape: a software environment for integrated models of biomolecular interaction networks. *Genome Res.* **13**, 2498-2504 (2003).

11 Su, G., Kuchinsky, A., Morris, J. H., States, D. J. & Meng, F. J. B. GLay: community structure analysis of biological networks. *Bioinformatics* **26**, 3135-3137 (2010).

12 Morris, J. H. *et al.* clusterMaker: a multi-algorithm clustering plugin for Cytoscape. *BMC Bioinformatics* **12**, 1-14 (2011).

13 VerHague, M. A., Cheng, D., Weinberg, R. B. & Shelness, G. S. Apolipoprotein A-IV expression in mouse liver enhances triglyceride secretion and reduces hepatic lipid content by promoting very low density lipoprotein particle expansion. *Arterioscler. Thromb. Vasc. Biol.* **33**, 2501-2508 (2013).

14 Tutusaus, A. *et al.* A Functional Role of GAS6/TAM in Nonalcoholic Steatohepatitis Progression Implicates AXL as Therapeutic Target. *Cell. Mol. Gastroenter.* **9**, 349-368 (2020).

15 Kurt, Z. *et al.* Tissue-specific pathways and networks underlying sexual dimorphism in non-alcoholic fatty liver disease. *Biol. Sex Differ.* **9**, 46 (2018).

16 Lian, C. Y., Zhai, Z. Z., Li, Z. F. & Wang, L. High fat diet-triggered non-alcoholic fatty liver disease: A review of proposed mechanisms. *Chem. Biol. Interact.* **330**, 109199 (2020).

17 Lefere, S., Devisscher, L. & Tacke, F. Targeting CCR2/5 in the treatment of nonalcoholic steatohepatitis (NASH) and fibrosis: opportunities and challenges. *Expert Opin. Investig. Drugs* **29**, 89-92 (2020).

18 Fang, M. *et al.* Abnormal CD44 activation of hepatocytes with nonalcoholic fatty accumulation in rat hepatocarcinogenesis. *World J. Gastrointest. Oncol.* **12**, 66-76 (2020).

19 Cazanave, S. *et al.* The Transcriptomic Signature Of Disease Development And Progression Of Nonalcoholic Fatty Liver Disease. *Sci. Rep.* **7**, 17193 (2017).

20 Hou, C. *et al.* Bioinformatics Analysis of Key Differentially Expressed Genes in Nonalcoholic Fatty Liver Disease Mice Models. *Gene Expr.* **19**, 25-35 (2018).

21 Zhou, L. *et al.* Cidea promotes hepatic steatosis by sensing dietary fatty acids. *Hepatology* **56**, 95-107 (2012).

22 Sans, A. *et al.* The Differential Expression of Cide Family Members is Associated with Nafld Progression from Steatosis to Steatohepatitis. *Sci. Rep.* **9**, 7501 (2019).

23 Fleet, T. *et al.* Genetic and Environmental Models of Circadian Disruption Link SRC-2 Function to Hepatic Pathology. *J. Biol. Rhythms* **31**, 443-460 (2016).

24 Heintz, M. M., Kumar, R., Rutledge, M. M. & Baldwin, W. S. Cyp2b-null male mice are susceptible to diet-induced obesity and perturbations in lipid homeostasis. *J. Nutr. Biochem.* **70**, 125-137 (2019).

25 Katayama, A. *et al.* Beneficial impact of Gpnmb and its significance as a biomarker in nonalcoholic steatohepatitis. *Sci. Rep.* **5**, 16920 (2015)

26 WATANABE, K. *et al.* ILDR2 Alters Hepatic Phospholipid Composition via MBOAT7—Effects on Liver Fat. *Diabetes* https://doi.org/10.2337/db18-1994-P (2018).

27 Herrera-Marcos, L. V. *et al.* Hepatic galectin-3 is associated with lipid droplet area in non-alcoholic steatohepatitis in a new swine model. *Sci. Rep.* **12**, 1024 (2022).

28 Naim, A., Pan, Q. & Baig, M. S. Matrix Metalloproteinases (MMPs) in Liver Diseases. *J. Clin. Exp. Hepatol.* **7**, 367-372 (2017).

29 Koop, A. C. *et al.* Therapeutic Targeting of Myeloperoxidase Attenuates NASH in Mice. *Hepatol. Commun.* **4**, 1441-1458 (2020).

30 Hsieh, J. *et al.* TTC39B deficiency stabilizes LXR reducing both atherosclerosis and steatohepatitis. *Nature* **535**, 303-307 (2016).
